# Supplementary material for: Decoding Chemotherapy Resistance of Undifferentiated Pleomorphic Sarcoma at the Single Cell Resolution: A Case Report
Source: J Clin Med. 2024 Nov 26;13(23):7176. doi: 10.3390/jcm13237176 (PMC11642494; doi:10.3390/jcm13237176)
Supplement: Supplementary file 1 [file jcm-13-07176-s001.zip › Supplementary Figures S1, S2 (track changes).pdf]

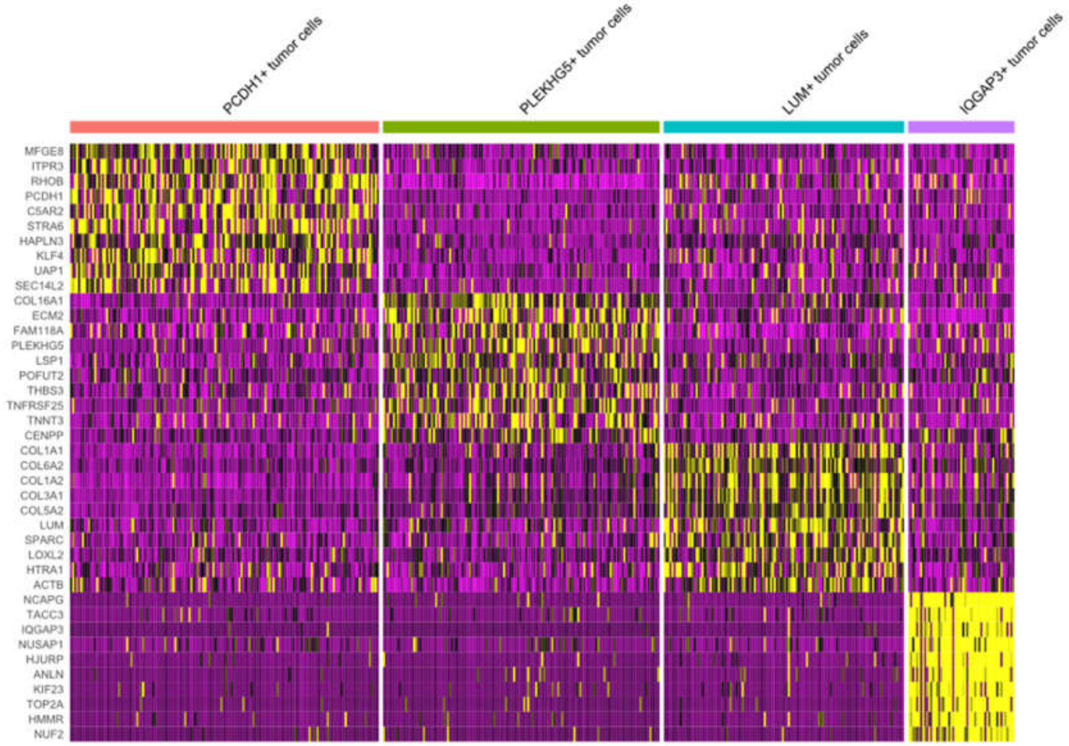

Figure S1. Heatmap of marker genes differentially expressed in tumor cell subpopulations.

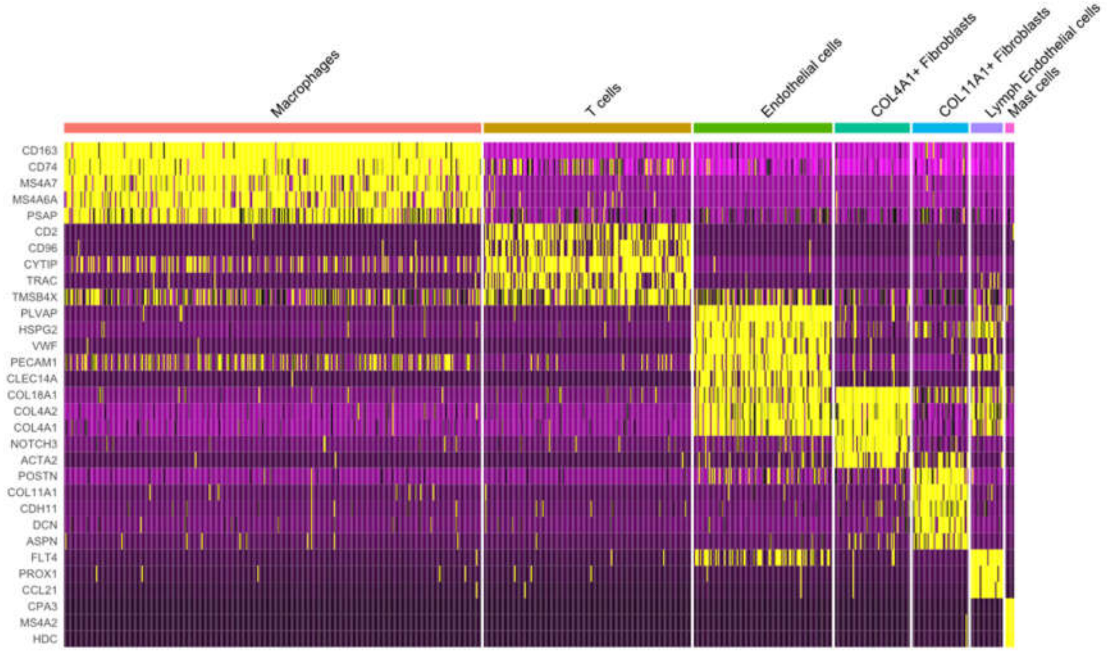

Figure S2. Heatmap of marker genes differentially expressed in TME cell types.
