# Supplementary material for: Decoding Chemotherapy Resistance of Undifferentiated Pleomorphic Sarcoma at the Single Cell Resolution: A Case Report
Source: J Clin Med. 2024 Nov 26;13(23):7176. doi: 10.3390/jcm13237176 (PMC11642494; doi:10.3390/jcm13237176)
Supplement: Supplementary file 1 [file jcm-13-07176-s001.zip › Supplementary Table S1 (track changes).pdf]

Supplementary Table S1. Differentially expressed genes in aneuploid tumor cell subpopulations, adjusted p value < 0.05.

PCDH1+ tumor cells		PLEKHG5+ tumor cells		LUM+ tumor cells		IQGAP3+ tumor cells	
Genes	LogFC	Genes	LogFC	Genes	LogFC	Genes	LogFC
MFGE8	1.51	*COL16A1*	1.29	*COL1A1*	1.50	*NCAPG*	6.25
ITPR3	1.94	*ECM2*	1.68	*COL6A2*	1.14	*TACC3*	4.82
RHOB	1.54	*FAM118A*	1.40	*COL1A2*	1.42	*IQGAP3*	7.57
PCDH1	2.76	*PLEKHG5*	1.97	*COL3A1*	1.36	*NUSAP1*	4.25
C5AR2	2.09	*LSP1*	1.04	*COL5A2*	1.03	*HJURP*	5.82
STRA6	2.75	*POFUT2*	1.10	*LUM*	1.75	*ANLN*	6.13
HAPLN3	1.56	*THBS3*	1.59	*SPARC*	1.25	*KIF23*	5.37
KLF4	1.94	*TNFRSF25*	1.84	*LOXL2*	1.24	*TOP2A*	6.28
UAP1	1.50	*TNNT3*	1.82	*HTRA1*	1.32	*HMMR*	5.57
SEC14L2	2.32	*CENPP*	1.00	*ACTB*	1.05	*NUF2*	5.82
ULK1	1.75	*PLPP1*	1.44	*BGN*	1.17	*CDK1*	5.48
SERPINE2	1.27	*MEIS1*	1.34	*C1R*	1.58	*TROAP*	7.30
ELN	1.35	*PODN*	1.01	*MMP2*	1.08	*FOXM1*	5.52
EPS8L2	1.84	*RNF138*	1.48	*MMP14*	1.15	*CIT*	5.30
CEBPB	1.10	*TNIK*	1.15	*CD63*	1.08	*CENPE*	5.00
JUNB	1.53	*IFITM10*	1.22	*ISLR*	1.21	*TPX2*	5.44
SORBS2	1.70	*MAST4*	1.09	*CAVIN1*	1.34	*MKI67*	5.17
ITGA5	1.19	*LIFR*	1.19	*LRRC15*	1.25	*CKAP2L*	5.12
DDIT4	1.10	*TANC1*	1.21	*TIMP1*	1.12	*KIFC1*	5.62
NGEF	1.56	*PER3*	1.04	*SERPINH1*	1.27	*CENPN*	4.45
SLC39A14	1.32	*ZNF608*	1.14	*MT-ND3*	1.28	*LMNB2*	3.25
LHFPL2	1.25	*ARHGEF19*	1.17	*COL4A1*	1.16	*PRC1*	4.69
IGFBP5	1.80	*NFATC2*	1.58	*COL4A2*	1.03	*CEP55*	5.99
PMP22	1.10	*MIB2*	1.03	*CFL1*	1.30	*CENPF*	5.71
AHNAK2	1.74	*ANKZF1*	1.02	*EMILIN1*	1.09	*CDCA2*	5.71
RDH10	1.67	*MUC1*	1.27	*GPNMB*	1.50	*BUB1*	5.22
TNFSF4	1.21	*CLK2*	1.05	*LGALS3BP*	1.00	*PBK*	5.87
A4GALT	1.51			*TCEAL8*	1.00	*HMGB2*	3.33
CLU	1.60			*CCDC85B*	1.32	*HIST1H1B*	5.96
ABI3BP	1.62			*DCN*	1.35	*GTSE1*	5.73

COMP	1.01
CHST15	1.32
ADAMTS17	1.23
ZNF365	1.47
ILDR2	1.27
ZFP36	1.16
TNS1	1.03
SLC29A1	1.11
AACS	1.13
C15orf39	1.45
TYRO3	1.25
MMP17	1.12
NPDC1	1.22
STK24	1.01
TNFAIP8L3	1.15
SCARA3	1.25
AK1	1.11
TNFAIP6	1.33
CEBPG	1.04
MAP3K8	1.07

PRSS23	1.45
ACTG1	1.30
PPIB	1.18
ATP5ME	1.25
MMP11	1.26
GRN	1.13
CTSB	1.18
GAS1	1.01
CKAP4	1.01
TM9SF3	1.02
SERPING1	1.21
FZD1	1.09
HSPA8	1.04
TCEAL9	1.02
FAM162A	1.01
TRAPPC5	1.06
UBA52	1.00

CDCA3	6.24
AURKB	6.74
SPAG5	3.24
NEIL3	6.47
SMC4	3.05
KIF20A	6.60
TTK	6.49
POLQ	4.48
SPC25	5.54
CENPI	3.62
SPC24	4.67
UBE2C	4.80
KIF15	5.22
CCNB1	5.87
KIF4A	5.16
KNL1	5.04
KIF18B	5.98
SOGA1	2.14
ASPM	5.68
BIRC5	5.79
DEPDC1B	4.16
CKAP2	4.57
DLGAP5	6.75
PHF19	2.37
KNSTRN	3.96
ZGRF1	3.07
NDC80	4.78
CDCA8	5.25
CKAP5	2.38
SGO2	4.05
CDKN2C	3.42
KIF22	2.79
MXD3	3.35

WDR62	3.85
CDC25C	5.43
LMNB1	3.23
UBE2T	4.75
NUCB2	2.44
KPNA2	3.28
KIF2C	5.71
HIST1H1C	3.22
KIF11	5.08
FZR1	2.13
RRM2	5.35
SCLT1	2.44
TICRR	5.04
CDC20	5.63
GAS2L3	5.01
CCDC18	2.85
KIF20B	4.13
C21orf58	3.44
CCNB2	3.94
ESPL1	5.16
CKS2	3.81
CCNA2	3.70
DIAPH3	3.80
NRP2	1.60
PARPBP	3.71
NEURL1B	2.96
CEP128	2.85
CENPW	3.78
PTTG1	4.12
EME1	3.62
CD82	2.30
CENPL	2.60
EZH2	2.73

CCNF	3.98
STIL	3.43
FAM83D	4.54
ASF1B	3.98
MND1	3.67
REEP4	3.48
ATAD5	2.57
BRIP1	3.23
NDE1	2.47
MIS18BP1	2.10
BRCA2	2.84
NCAPD2	3.50
AC091057.6	3.77
RECQL4	3.56
CENPU	3.00
FANCI	3.01
UHRF1	2.51
CCDC150	3.20
FBXO5	3.61
CLEC11A	3.60
TUBB	2.29
MELK	3.66
CENPM	3.17
KMT5A	2.52
MGME1	2.66
NCAPD3	2.14
STMN3	2.47
TMPO	2.44
UBALD2	3.23
CIP2A	2.99
CENPO	2.99
NSD2	1.80
NEMP1	2.46

ZNF724	3.12
NAV2	2.56
RFX8	1.69
ARHGAP33	1.92
RAD54L	3.07
PTMS	1.82
CENPK	1.72
ANP32E	2.07
XRCC2	2.83
CDCA4	2.64
BRD8	1.54
NCAPG2	1.82
DBF4B	2.48
SUN2	1.76
MPHOSPH9	2.01
CEP72	2.44
HP1BP3	1.72
GGH	2.42
HIST1H1E	2.20
RANGAP1	2.19
HDGF	1.88
DCK	2.16
CENPH	2.14
DCAF7	1.28
POLH	2.15
TEDC1	2.33
CALM2	1.52
GINS1	2.31
TCF19	2.37
FOSL2	1.24
C1orf112	1.76
GEN1	2.07
CEP97	1.58

DDX39A	1.53
MYO10	1.30
C9orf40	1.96
CEP85	2.19
FAM122B	1.43
RCCD1	1.75
CEP192	1.53
PPP2R3B	1.48
LGALS1	1.78
GJC1	1.88
CNTROB	1.56
C2orf69	1.96
FAM111A	1.33
NUCKS1	1.82
LIG1	1.91
SMAD3	1.37
BUB3	1.58
CENPJ	1.78
VRK1	1.66
FGFR1OP	1.56
SMTN	1.30
CA5B	1.35
CALM3	1.64
LIN54	1.96
MTBP	1.67
CNTRL	1.08
ZNF519	1.78
MASTL	1.89
SPIN4	1.42
SLC25A25	1.99
PCNT	1.02
CBX5	1.26
DIXDC1	1.20

SPATA5	1.41
PPP2R5C	1.36
KIF5B	1.47
FANCA	1.39
C5orf34	1.91
G2E3	1.52
H1FX	1.46
SMC2	1.63
POLE	1.54
SAP30	1.29
RFWD3	1.77
TUBB6	1.58
PKN3	1.47
RAD18	1.62
HAUS3	1.24
ZNF69	1.47
DCTN3	1.22
DCAF15	1.51
