# Supplementary material for: Decoding Chemotherapy Resistance of Undifferentiated Pleomorphic Sarcoma at the Single Cell Resolution: A Case Report
Source: J Clin Med. 2024 Nov 26;13(23):7176. doi: 10.3390/jcm13237176 (PMC11642494; doi:10.3390/jcm13237176)
Supplement: Supplementary file 1 [file jcm-13-07176-s001.zip › Supplementary Table S2 (track changes).pdf]

Supplementary Table S2. Signaling pathways (KEGG) and biological processes (GO) enriched in tumor cell subpopulations, adjusted p value < 0.05.

PCDH1+ tumor cells			
KEGG Term	Genes	GO Term	Genes
		Integrated Stress Response Signaling (GO:0140467)	*CEBPB;CEBPG;JUNB*
PLEKHG5+ tumor cells			
LUM+ tumor cells			
Proteoglycans in cancer	*FZD1;COL1A1;CD63;COL1A2;LUM;MMP2;ACTB;DCN;ACTG1*	Extracellular Matrix Organization (GO:0030198)	*COL1A1;MMP11;COL3A1;MMP14;COL1A2;COL4A2;LUM;COL4A1;MMP2;COL5A2;SERPINH1;LOXL2*
Protein digestion and absorption	*COL1A1;COL3A1;COL1A2;COL4A2;COL4A1;COL6A2;COL5A2*	Extracellular Structure Organization (GO:0043062)	*COL1A1;MMP11;COL3A1;MMP14;COL1A2;COL4A2;COL4A1;MMP2;COL5A2*
AGE-RAGE signaling pathway in diabetic complications	*COL1A1;COL3A1;COL1A2;COL4A2;COL4A1;MMP2*	External Encapsulating Structure Organization (GO:0045229)	*COL1A1;MMP11;COL3A1;MMP14;COL1A2;COL4A2;COL4A1;MMP2;COL5A2*
Focal adhesion	*COL1A1;COL1A2;COL4A2;COL4A1;COL6A2;ACTB;ACTG1*	Collagen Fibril Organization (GO:0030199)	*COL1A1;COL3A1;COL1A2;LUM;COL5A2;SERPINH1;LOXL2*
Relaxin signaling pathway	*COL1A1;COL3A1;COL1A2;COL4A2;COL4A1;MMP2*	Supramolecular Fiber Organization (GO:0097435)	*COL1A1;COL3A1;COL1A2;LUM;COL5A2;SERPINH1;EMILIN1;LOXL2*
ECM-receptor interaction	*COL1A1;COL1A2;COL4A2;COL4A1;COL6A2*	Regulation Of Angiogenesis (GO:0045765)	*GRN;SPARC;GPNMB;COL4A2;EMILIN1;DCN*
Amoebiasis	*COL1A1;COL3A1;COL1A2;COL4A2;COL4A1*	Negative Regulation Of Peptidase Activity (GO:0010466)	*SERPINH1;SERPING1;TIMP1;CTSB*
Platelet activation	*COL1A1;COL3A1;COL1A2;ACTB;ACTG1*	Positive Regulation Of Cell Motility (GO:2000147)	*COL1A1;LRRC15;GRN;MMP14;GPNMB;CAVIN1*

Human papillomavirus infection	*FZD1;COL1A1;COL1A2;COL4A2;COL4A1;COL6A2*	Skin Development (GO:0043588)	*COL1A1;COL3A1;COL1A2;COL5A2*
Pertussis	*C1R;CFL1;SERPING1*	Negative Regulation Of Blood Vessel Morphogenesis (GO:2000181)	*SPARC;COL4A2;EMILIN1;DCN*
Diabetic cardiomyopathy	*COL1A1;COL3A1;COL1A2;MMP2*	Negative Regulation Of Angiogenesis (GO:0016525)	*SPARC;COL4A2;EMILIN1;DCN*
PI3K-Akt signaling pathway	*COL1A1;COL1A2;COL4A2;COL4A1;COL6A2*	Endodermal Cell Differentiation (GO:0035987)	*MMP14;COL4A2;MMP2*
Leukocyte transendothelial migration	*MMP2;ACTB;ACTG1*	Regulation Of Cell Migration (GO:0030334)	*COL1A1;LRRC15;GRN;MMP14;GPNMB;EMILIN1;ACTG1*
Fluid shear stress and atherosclerosis	*MMP2;ACTB;ACTG1*	Endoderm Formation (GO:0001706)	*MMP14;COL4A2;MMP2*
Apoptosis	*ACTB;ACTG1;CTSB*	Negative Regulation Of Proteolysis (GO:0045861)	*GAS1;TIMP1;CTSB*
Phagosome	*C1R;ACTB;ACTG1*	Regulation Of Defense Response To Bacterium (GO:1900424)	*GRN;EMILIN1*
Vibrio cholerae infection	*ACTB;ACTG1*	Skin Morphogenesis (GO:0043589)	*COL1A1;COL1A2*
Hippo signaling pathway	*FZD1;ACTB;ACTG1*	Extracellular Matrix Disassembly (GO:0022617)	*MMP11;MMP14;MMP2*
Hepatocellular carcinoma	*FZD1;ACTB;ACTG1*	Cellular Component Disassembly (GO:0022411)	*MMP11;MMP14;MMP2*

Viral myocarditis	*ACTB;ACTG1*	Negative Regulation Of Multicellular Organismal Process (GO:0051241)	*GRN;GPNMB;EMILIN1;TIMP1;LOXL2*
Adherens junction	*ACTB;ACTG1*	Cellular Response To UV-A (GO:0071492)	*MMP2;TIMP1*
Gastric acid secretion	*ACTB;ACTG1*	Positive Regulation Of Defense Response To Bacterium (GO:1900426)	*GRN;EMILIN1*
Arrhythmogenic right ventricular cardiomyopathy	*ACTB;ACTG1*	Skeletal System Development (GO:0001501)	*COL1A1;MMP14;COL1A2;PPIB*
Bacterial invasion of epithelial cells	*ACTB;ACTG1*	Response To UV-A (GO:0070141)	*MMP2;TIMP1*
Antigen processing and presentation	*HSPA8;CTSB*	Positive Regulation Of Cell Migration (GO:0030335)	*COL1A1;LRRC15;GRN;MMP14;GPNMB*
Regulation of actin cytoskeleton	*CFL1;ACTB;ACTG1*	Negative Regulation Of Endopeptidase Activity (GO:0010951)	*SERPINH1;SERPING1;TIMP1*
		Regulation Of Endopeptidase Activity (GO:0052548)	*SERPINH1;SERPING1;TIMP1*
		Positive Regulation By Host Of Viral Process (GO:0044794)	*CFL1;PPIB*
		Regulation Of Protein Localization To	*LRRC15;MMP14;ACTB*

Plasma Membrane (GO:1903076)	
Regulation Of Integrin-Mediated Signaling Pathway (GO:2001044)	*CD63;TIMP1*
Regulation Of Vascular Endothelial Growth Factor Signaling Pathway (GO:1900746)	*CD63;DCN*
Regulation Of Protein Localization To Cell Periphery (GO:1904375)	*MMP14;ACTB*
Regulation Of Endothelial Cell Migration (GO:0010594)	*GRN;SPARC;DCN*
Regulation Of Apoptotic Process (GO:0042981)	*FAM162A;CFL1;HTRA1;GAS1;EMILIN1;ACTB;CTSB*
Modulation By Host Of Viral Process (GO:0044788)	*CFL1;PPIB*
Positive Regulation Of Multicellular Organismal Process (GO:0051240)	*COL1A1;CFL1;PPIB;ACTB;LOXL2*
Cell-Matrix Adhesion (GO:0007160)	*CD63;COL3A1;EMILIN1*

		Regulation Of Cell Morphogenesis (GO:0022604)	*SPARC;CFL1*
		Regulation Of Anatomical Structure Morphogenesis (GO:0022603)	*SPARC;GPNMB;CFL1*
		Positive Regulation Of Apoptotic Process (GO:0043065)	*GRN;FAM162A;HTRA1;EMILIN1*
		Odontogenesis (GO:0042476)	*COL1A1;COL1A2*
		Positive Regulation Of Response To Biotic Stimulus (GO:0002833)	*GRN;EMILIN1*
IQGAP3$^+$ tumor cells			
Cell cycle	*SMAD3;CDKN2C;TTK;CDC25C;CDC20;CCNA2;CCNB2;CCNB1;FZR1;PTTG1;ESPL1;CDK1;BUB3;BUB1*	Mitotic Sister Chromatid Segregation (GO:0000070)	*NCAPG2;CDCA8;NCAPG;KIF11;SMC4;SMC2;CCNB1;NUSAP1;CEP192;KMT5A;CEP55;DLGAP5;SPAG5;KIF23;KIF22;KNSTRN;NDC80;TPX2;CENPE;KIF18B;ESPL1;KIFC1;CENPI;PRC1;CENPK;KIF2C;NCAPD2;NCAPD3*
Oocyte meiosis	*CDC20;CCNB2;CCNB1;PTTG1;ESPL1;CDK1;CALM3;FBXO5;PPP2R5C;CDC25C;CALM2;BUB1*	Microtubule Cytoskeleton Organization Involved In Mitosis (GO:1902850)	*SUN2;STIL;CDCA8;TTK;KIF11;WDR62;CKAP5;AURKB;NDC80;CENPE;CCNB1;ESPL1;CENPH;KIF4A;NUF2;NUSAP1;CDK1;TACC3;BIRC5;PCNT;DLGAP5;SPC25*
Fanconi anemia pathway	*FANCI;BRIP1;EME1;UBE2T;FANCA;BRCA2;POLH*	Mitotic Spindle Organization (GO:0007052)	*SUN2;STIL;CDCA8;KIF23;TTK;KIF11;WDR62;CKAP5;AURKB;NDC80;CENPE;TPX2;CCNB1;CENPH;KIF*

			C1;PRC1;KIF4A;NUF2;BIRC5;CEP192;PCNT;DLGAP5;SPC25
Progesterone-mediated oocyte maturation	*CCNA2;CCNB2;CCNB1;FZR1;CDK1;KIF22;CDC25C;BUB1*	Positive Regulation Of Cell Cycle Process (GO:0090068)	*GEN1;STIL;SPAG5;NCAPG2;CDCA8;NCAPG;KIF23;DBF4B;SMC4;NDC80;AURKB;SMC2;CIT;CENPJ;NUSAP1;BIRC5;NCAPD2;NCAPD3;KIF20B;RAD18*
Cellular senescence	*CCNA2;CCNB2;LIN54;CCNB1;SMAD3;CDK1;CALM3;FOXM1;CALM2*	Sister Chromatid Segregation (GO:0000819)	*TOP2A;KIF18B;SPAG5;ESPL1;CENPI;KIFC1;NCAPG2;CENPK;NUSAP1;SMC4;KNSTRN;NDC80*
Homologous recombination	*BRIP1;EME1;XRCC2;RAD54L;BRCA2*	Mitotic Nuclear Division (GO:0140014)	*SPAG5;UBE2C;NDE1;NCAPG2;SMC4;KNSTRN;NDC80;KIF18B;ESPL1;CENPI;KIFC1;CENPK;NUSAP1*
p53 signaling pathway	*CCNB2;CCNB1;RRM2;CD82;CDK1;GTSE1*	Regulation Of Chromosome Segregation (GO:0051983)	*CDCA2;NCAPG2;NCAPG;KIF2C;NCAPD2;NCAPD3;MKI67;SMC4;BUB1;RAD18;SMC2*
Human T-cell leukemia virus 1 infection	*CDC20;CCNA2;CCNB2;CDKN2C;SMAD3;PTTG1;ESPL1;BUB3*	Mitotic Cytokinesis (GO:0000281)	*CKAP2;CDCA8;KIF23;AURKB;CIT;ANLN;ESPL1;KIF4A;NUSAP1;BIRC5;KIF20A;CNTROB;CEP55*
		Regulation Of Cell Cycle Process (GO:0010564)	*CDCA2;SPAG5;KIF11;DBF4B;MKI67;CDC25C;KNSTRN;AURKB;PRC1;CALM3;KIF2C;FBXO5;KIF20A;CEP85;KIF20B;CALM2;BUB1*
		Positive Regulation Of Chromosome Segregation (GO:0051984)	*CCNB1;NCAPG2;CDK1;NCAPG;NCAPD2;NCAPD3;SMC4;RAD18;SMC2*
		Negative Regulation Of Mitotic Metaphase/Anaphase Transition (GO:0045841)	*CDC20;CENPF;NUF2;TTK;BUB3;FBXO5;BUB1;NDC80;SPC24;SPC25*

Positive Regulation Of Chromosome Separation (GO:1905820)	*NCAPG2;CDCA8;NCAPG;BIRC5;NCAPD2;NCAPD3;SMC4;SMC2*
DNA Repair (GO:0006281)	*FANCI;POLQ;MGME1;LIG1;ZGRF1;XRCC2;FANCA;NUCKS1;BRCA2;TICRR;RECQL4;FAM111A;NEIL3;DEPDC1B;EME1;RFWD3;UBE2T;NSD2;CDK1;POLE;RAD18;POLH*
Positive Regulation Of Mitotic Cell Cycle Phase Transition (GO:1901992)	*CDC20;CCNB1;STIL;RRM2;ESPL1;UBE2C;CENPJ;CDK1;FBXO5;DBF4B;CDC25C;DLGAP5*
Cytoskeleton-Dependent Cytokinesis (GO:0061640)	*DCTN3;CKAP2;CDCA8;KIF23;AURKB;CIT;ANLN;ESPL1;KIF4A;NUSAP1;BIRC5;KIF20A;CEP55*
Spindle Assembly Checkpoint Signaling (GO:0071173)	*CDC20;CENPF;NUF2;TTK;BUB3;BUB1;NDC80;SPC24;SPC25*
Mitotic Spindle Assembly Checkpoint Signaling (GO:0007094)	*CDC20;CENPF;NUF2;TTK;BUB3;BUB1;NDC80;SPC24;SPC25*
Mitotic Spindle Checkpoint Signaling (GO:0071174)	*CDC20;CENPF;NUF2;TTK;BUB3;BUB1;NDC80;SPC24;SPC25*
Mitotic Spindle Assembly (GO:0090307)	*TPX2;KIFC1;PRC1;KIF4A;CDCA8;BIRC5;CEP192;KIF23;KIF11;AURKB*
Positive Regulation Of Mitotic Sister	*CDC20;ESPL1;UBE2C;CDCA8;BIRC5;AURKB;DLGAP5*

Chromatid Separation (GO:1901970)	
Mitotic Chromosome Condensation (GO:0007076)	*NUSAP1;NCAPG;NCAPD2;KMT5A;NCAPD3;SMC4;SMC2*
Metaphase Plate Congression (GO:0051310)	*CENPE;CCNB1;CENPF;KIFC1;CDCA8;KIF2C;KIF22;FAM83D;CEP55;NDC80*
Kinetochore Organization (GO:0051383)	*CENPE;CENPF;CENPW;CENPH;NUF2;DLGAP5*
Regulation Of Chromosome Separation (GO:1905818)	*NCAPG2;NCAPG;NCAPD2;NCAPD3;SMC4;SMC2*
Chromosome Condensation (GO:0030261)	*TOP2A;NUSAP1;NCAPG;NCAPD2;KMT5A;NCAPD3;SMC4;SMC2*
Positive Regulation Of Chromosome Organization (GO:2001252)	*NCAPG2;CDK1;NCAPG;NCAPD2;NCAPD3;SMC4;AURKB;SMC2*
DNA Metabolic Process (GO:0006259)	*TOP2A;GEN1;POLQ;LIG1;XRCC2;FANCA;HMGB2;PTMS;TICRR;RECQL4;FAM111A;NEIL3;UBE2T;CDK1;KPNA2;POLE;RAD18;POLH*
Mitotic Cell Cycle Phase Transition (GO:0044772)	*CCNA2;CCNB2;CCNB1;MELK;UBE2C;CCNF;CDK1;TACC3;CDC25C;FOXM1;MASTL;POLE*
Mitotic Metaphase Plate Congression (GO:0007080)	*CENPE;CCNB1;KIFC1;NUF2;CDCA8;KIF2C;KIF22;CEP55;NDC80*

Regulation Of Chromosome Condensation (GO:0060623)	*NCAPG2;NCAPG;NCAPD3;SMC4;SMC2*
Positive Regulation Of Chromosome Condensation (GO:1905821)	*NCAPG2;NCAPG;NCAPD3;SMC4;SMC2*
Centromere Complex Assembly (GO:0034508)	*CENPE;CENPF;CENPW;CENPI;HJURP;DLGAP5*
Establishment Of Chromosome Localization (GO:0051303)	*CENPF;NDE1;KIF2C;KIF22;FAM83D;NDC80*
Cell Cycle G2/M Phase Transition (GO:0044839)	*CCNA2;CCNB1;MELK;CDK1;FOXM1;CDC25C;MASTL;AURKB*
Nuclear Chromosome Segregation (GO:0098813)	*TOP2A;CENPF;KIF2C;KIF22;FAM83D;NDC80*
Regulation Of Mitotic Cell Cycle Spindle Assembly Checkpoint (GO:0090266)	*GEN1;CCNB1;CDCA8;BIRC5;AURKB;NDC80*
Protein Localization To Condensed Chromosome (GO:1903083)	*MTBP;CDK1;TTK;BUB3;KNL1*
Mitotic Spindle Elongation (GO:0000022)	*PRC1;KIF4A;BIRC5;CDCA8;KIF23*

Mitotic Spindle Midzone Assembly (GO:0051256)	*PRC1;KIF4A;BIRC5;CDCA8;KIF23*
Protein Localization To Kinetochore (GO:0034501)	*MTBP;CDK1;TTK;BUB3;KNL1*
G2/M Transition Of Mitotic Cell Cycle (GO:0000086)	*CCNA2;CCNB1;MELK;CDK1;FOXM1;CDC25C;MASTL*
DNA Damage Response (GO:0006974)	*TOP2A;POLQ;CBX5;LIG1;XRCC2;FANCA;VRK1;MASTL;TICRR;RECQL4;FAM111A;DEPDC1B;RFWD3;UBE2T;CDK1;FBXO5;RAD18;POLH*
Double-Strand Break Repair (GO:0006302)	*GEN1;RECQL4;POLQ;DEPDC1B;EME1;ZGRF1;XRCC2;RFWD3;NSD2;HMGB2;NUCKS1;BRCA2*
Regulation Of Mitotic Metaphase/Anaphase Transition (GO:0030071)	*CDC20;CENPE;CCNB1;ESPL1;UBE2C;FBXO5;DLGAP5*
Regulation Of Mitotic Cell Cycle (GO:0007346)	*CDC20;CCNB1;FZR1;CDCA2;CKS2;FBXO5;MKI67;CDC25C;KIF20B;DLGAP5*
Protein Localization To Chromosome, Centromeric Region (GO:0071459)	*MTBP;CDK1;TTK;KNL1;BUB3*
Regulation Of Nuclear Division (GO:0051783)	*CDCA2;FBXO5;CDC25C;MKI67;KIF20B*
Kinetochore Assembly (GO:0051382)	*CENPE;CENPF;CENPW;DLGAP5*

Regulation Of G2/M Transition Of Mitotic Cell Cycle (GO:0010389)	*CCNB1;CENPF;CDK1;DBF4B;FBXO5;CDC25C*
Regulation Of Mitotic Nuclear Division (GO:0007088)	*CCNB1;CDCA2;NUSAP1;FBXO5;MKI67;CDC25C;KIF20B*
Regulation Of Cytokinesis (GO:0032465)	*PRC1;KIF23;CALM3;KIF20A;KIF20B;CALM2;AURKB;CIT*
Positive Regulation Of G2/M Transition Of Mitotic Cell Cycle (GO:0010971)	*CCNB1;CDK1;DBF4B;FBXO5;CDC25C*
Regulation Of Mitotic Sister Chromatid Separation (GO:0010965)	*CCNB1;BIRC5;CDCA8;AURKB*
Regulation Of Protein Serine/Threonine Kinase Activity (GO:0071900)	*CCNA2;CCNB2;CCNB1;CDKN2C;CCNF;CALM3;CDC25C;CALM2;EZH2*
Positive Regulation Of Metaphase/Anaphase Transition Of Cell Cycle (GO:1902101)	*CDC20;ESPL1;UBE2C;DLGAP5*
Positive Regulation Of Mitotic Cell Cycle Spindle Assembly Checkpoint (GO:0090267)	*GEN1;BIRC5;CDCA8;NDC80*
Positive Regulation Of Mitotic	*CDC20;ESPL1;UBE2C;DLGAP5*

Metaphase/Anaphase Transition (GO:0045842)	
Positive Regulation Of Spindle Checkpoint (GO:0090232)	*GEN1;BIRC5;CDCA8;NDC80*
Positive Regulation Of Cell Cycle G2/M Phase Transition (GO:1902751)	*CCNB1;CDK1;DBF4B;FBXO5;CDC25C*
Attachment Of Mitotic Spindle Microtubules To Kinetochore (GO:0051315)	*CENPE;NUF2;KIF2C;NDC80*
DNA-templated DNA Replication Maintenance Of Fidelity (GO:0045005)	*GEN1;FAM111A;EME1;RFWD3;NUCKS1;POLE*
Replication Fork Processing (GO:0031297)	*GEN1;FAM111A;EME1;RFWD3;NUCKS1;BRCA2*
Positive Regulation Of Mitotic Cytokinesis (GO:1903490)	*BIRC5;CDCA8;KIF20B*
Regulation Of Cyclin-Dependent Protein Kinase Activity (GO:1904029)	*CCNA2;CCNB2;CCNB1;CDKN2C;CCNF;CDC25C*
CENP-A Containing Chromatin Assembly (GO:0034080)	*CENPW;CENPI;HJURP*

Regulation Of Mitotic Cytokinesis (GO:1902412)	*BIRC5;CDCA8;AURKB*
Chromatin Remodeling At Centromere (GO:0031055)	*CENPW;CENPI;HJURP*
Nuclear Envelope Organization (GO:0006998)	*REEP4;NEMP1;LMNB2;LMNB1*
Regulation Of Ubiquitin Protein Ligase Activity (GO:1904666)	*CDC20;FZR1;UBE2C;FBXO5*
Microtubule Depolymerization (GO:0007019)	*KIF18B;STMN3;KIF2C;CKAP5*
Mitotic DNA Damage Checkpoint Signaling (GO:0044773)	*DEPDC1B;FZR1;EME1;RFWD3;CDK1;TICRR*
Positive Regulation Of Mitotic Nuclear Division (GO:0045840)	*CDC20;ESPL1;UBE2C;NUSAP1;DLGAP5*
Interstrand Cross-Link Repair (GO:0036297)	*FANCI;NEIL3;RFWD3;FANCA;NUCKS1*
Regulation Of Spindle Organization (GO:0090224)	*TPX2;STIL;CENPJ;TACC3*
Regulation Of Mitotic Spindle Organization (GO:0060236)	*TPX2;STIL;CENPJ;TACC3;CEP97*

Positive Regulation Of Cytokinesis (GO:0032467)	*CDCA8;BIRC5;KIF23;KIF20B;CIT*
Heterochromatin Organization (GO:0070828)	*UHRF1;HP1BP3;LMNB2;LMNB1;EZH2*
Positive Regulation Of Cell Cycle G1/S Phase Transition (GO:1902808)	*STIL;RRM2;CENPJ;FAM83D;EZH2*
Regulation Of Mitotic Sister Chromatid Segregation (GO:0033047)	*CCNB1;CDK1;AURKB*
Positive Regulation Of Ubiquitin Protein Ligase Activity (GO:1904668)	*CDC20;FZR1;UBE2C*
Regulation Of Attachment Of Spindle Microtubules To Kinetochore (GO:0051988)	*CCNB1;SPAG5;KNSTRN*
Chromosome Organization (GO:0051276)	*TOP2A;CENPW;CENPH;NUF2;CDCA8*
DNA Replication (GO:0006260)	*RECQL4;FAM111A;CDK1;POLE;PTMS;TICRR*
Positive Regulation Of Attachment Of Spindle Microtubules To Kinetochore (GO:0051987)	*CCNB1;BIRC5;CDCA8*

Microtubule Polymerization Or Depolymerization (GO:0031109)	*KIF18B;STMN3;KIF2C;CKAP5*
Regulation Of Cell Cycle Checkpoint (GO:1901976)	*DEPDC1B;RFWD3;BRCA2*
Regulation Of Cyclin-Dependent Protein Serine/Threonine Kinase Activity (GO:0000079)	*CCNA2;CCNB2;CCNB1;CDKN2C;CCNF;CDC25C*
Negative Regulation Of Gene Expression, Epigenetic (GO:0045814)	*UHRF1;LMNB2;LMNB1;EZH2;PHF19*
Protein Depolymerization (GO:0051261)	*KIF18B;STMN3;KIF2C;CKAP5*
Regulation Of Spindle Assembly (GO:0090169)	*STIL;SPAG5;CENPJ;CEP97*
Histone Monoubiquitination (GO:0010390)	*DEPDC1B;UHRF1;BRCA2*
Regulation Of Cell Division (GO:0051302)	*KIF18B;PRC1;CALM3;KIF20A;CALM2;AURKB*
Mitotic G2 DNA Damage Checkpoint Signaling (GO:0007095)	*FZR1;DEPDC1B;CDK1;TICRR*

Regulation Of Centrosome Duplication (GO:0010824)	*GEN1;STIL;CENPJ;CCNF*
Positive Regulation Of G1/S Transition Of Mitotic Cell Cycle (GO:1900087)	*MTBP;STIL;RRM2;CENPJ*
Heterochromatin Formation (GO:0031507)	*UHRF1;LMNB2;LMNB1;EZH2*
Regulation Of Cell Cycle (GO:0051726)	*CDC20;FZR1;CCNF;CKS2;PPP2R3B;FBXO5;BRD8;KIF20B;FOXM1;MASTL;DLGAP5*
protein-DNA Complex Organization (GO:0071824)	*CBX5;HMGB2;NUCKS1;EZH2;PHF19*
Establishment Of Mitotic Spindle Localization (GO:0040001)	*ESPL1;NDE1;NUSAP1;NDC80*
Microtubule Polymerization (GO:0046785)	*TPX2;NDE1;CENPJ;CKAP5*
Protein Localization To Microtubule Organizing Center (GO:1905508)	*STIL;SPAG5;CEP192*
Recombinational Repair (GO:0000725)	*GEN1;RECQL4;XRCC2;RFWD3;NUCKS1;BRCA2*
Base-Excision Repair (GO:0006284)	*POLQ;NEIL3;LIG1;POLE*

Nuclear Migration (GO:0007097)	*SUN2;LMNB2;LMNB1*
Negative Regulation Of Ubiquitin-Protein Transferase Activity (GO:0051444)	*CDC20;BUB3;FBXO5*
Protein Localization To Centrosome (GO:0071539)	*STIL;SPAG5;CEP192*
Regulation Of DNA Damage Checkpoint (GO:2000001)	*DEPDC1B;RFWD3;BRCA2*
Protein Autoubiquitination (GO:0051865)	*DEPDC1B;UHRF1;UBE2T;BRCA2;RAD18*
Nucleus Organization (GO:0006997)	*REEP4;LMNB2;CEP55;LMNB1*
Double-Strand Break Repair Via Homologous Recombination (GO:0000724)	*GEN1;RECQL4;XRCC2;RFWD3;NUCKS1;BRCA2*
Negative Regulation Of Double-Strand Break Repair Via Homologous Recombination (GO:2000042)	*POLQ;PARPBP;KMT5A*
Anaphase-Promoting Complex-Dependent Catabolic Process (GO:0031145)	*CDC20;FZR1;UBE2C*

Mitotic G2/M Transition Checkpoint (GO:0044818)	*FZR1;DEPDC1B;CDK1;TICRR*
Protein Monoubiquitination (GO:0006513)	*UHRF1;UBE2T;FANCA;RAD18*
Positive Regulation Of Spindle Assembly (GO:1905832)	*STIL;SPAG5*
Spindle Assembly (GO:0051225)	*TPX2;KIFC1;HAUS3;CEP192;KIF11*
Microtubule Nucleation (GO:0007020)	*TPX2;NDE1;CENPJ*
Regulation Of DNA Metabolic Process (GO:0051052)	*CCNA2;DEPDC1B;NUCKS1;FBXO5;KPNA2;POLH*
Regulation Of Mitotic Cell Cycle Phase Transition (GO:1901990)	*ANLN;CENPE;CENPF;CDKN2C;UBE2C*
Regulation Of Cyclic-Nucleotide Phosphodiesterase Activity (GO:0051342)	*CALM3;CALM2*
Positive Regulation Of Attachment Of Mitotic Spindle Microtubules To Kinetochore (GO:1902425)	*BIRC5;CDCA8*
Positive Regulation Of Ryanodine-Sensitive	*CALM3;CALM2*

Calcium-Release Channel Activity (GO:0060316)	
Regulation Of Attachment Of Mitotic Spindle Microtubules To Kinetochore (GO:1902423)	*BIRC5;CDCA8*
Positive Regulation Of Ubiquitin-Protein Transferase Activity (GO:0051443)	*CDC20;FZR1;UBE2C*
DNA Topological Change (GO:0006265)	*TOP2A;HMGB2*
Regulation Of Sister Chromatid Cohesion (GO:0007063)	*ESPL1;BUB1*
Negative Regulation Of Chromosome Organization (GO:2001251)	*TOP2A;ESPL1*
Positive Regulation Of Dephosphorylation (GO:0035306)	*CDCA2;CALM3;CALM2*
Protein K11-linked Ubiquitination (GO:0070979)	*FZR1;UBE2C;UBE2T*
Protein Localization To Microtubule Cytoskeleton (GO:0072698)	*FAM83D;KIF20B*

Protein Localization To Nuclear Envelope (GO:0090435)	*LMNB2;LMNB1*
Protein Polymerization (GO:0051258)	*DIAPH3;UBE2C;CENPJ;CKAP5*
Positive Regulation Of Protein Kinase Activity (GO:0045860)	*CCNA2;TPX2;CENPE;CALM3;CALM2;EZH2*
Regulation Of Ubiquitin-Protein Transferase Activity (GO:0051438)	*CDC20;BUB3;FBXO5*
Mitotic DNA Replication (GO:1902969)	*GINS1;LIG1*
Negative Regulation Of Double-Strand Break Repair (GO:2000780)	*POLQ;PARPBP;KMT5A*
Positive Regulation Of Protein Dephosphorylation (GO:0035307)	*CDCA2;CALM3;CALM2*
Negative Regulation Of Cell Cycle Process (GO:0010948)	*ESPL1;FBXO5;AURKB*
Regulation Of Cellular Response To Stress (GO:0080135)	*DEPDC1B;RFWD3;KMT5A;BRCA2;POLH*
Negative Regulation Of Release Of	*CALM3;CALM2*

Sequestered Calcium Ion Into Cytosol (GO:0051280)	
Negative Regulation Of Ryanodine-Sensitive Calcium-Release Channel Activity (GO:0060315)	*CALM3;CALM2*
Response To X-ray (GO:0010165)	*DEPDC1B;NUCKS1*
Histone H2A Ubiquitination (GO:0033522)	*DEPDC1B;BRCA2*
Regulation Of Cell Communication By Electrical Coupling Involved In Cardiac Conduction (GO:1901844)	*CALM3;CALM2*
DNA-templated DNA Replication (GO:0006261)	*RECQL4;GINS1;MGME1;POLE*
DNA Conformation Change (GO:0071103)	*TOP2A;HMGB2*
V(D)J Recombination (GO:0033151)	*LIG1;HMGB2*
Base-Excision Repair, Gap-Filling (GO:0006287)	*LIG1;POLE*
Detection Of Calcium Ion (GO:0005513)	*CALM3;CALM2*

Regulation Of Cell Communication By Electrical Coupling (GO:0010649)	*CALM3;CALM2*
Regulation Of Double-Strand Break Repair Via Homologous Recombination (GO:0010569)	*POLQ;PARPBP;KMT5A;BRD8*
Peptidyl-Serine Phosphorylation (GO:0018105)	*PKN3;CDK1;PBK;VRK1;TTK;MASTL*
Negative Regulation Of Transferase Activity (GO:0051348)	*CDC20;BUB3;FBXO5*
Regulation Of Phosphoprotein Phosphatase Activity (GO:0043666)	*CALM3;MASTL;CALM2*
Response To Ionizing Radiation (GO:0010212)	*DEPDC1B;RFWD3;BRCA2;TICRR*
Positive Regulation Of Intracellular Transport (GO:0032388)	*SPAG5;PCNT;KIF20B*
Regulation Of DNA Replication (GO:0006275)	*CCNA2;ATAD5;NUCKS1;FBXO5*
Regulation Of Protein Dephosphorylation (GO:0035304)	*CDCA2;CALM3;CALM2*

Peptidyl-Serine Modification (GO:0018209)	*PKN3;CDK1;PBK;VRK1;TTK;MASTL*
Membrane Organization (GO:0061024)	*REEP4;KIF20A;NEMP1;LMNB2;CEP55;LMNB1*
Regulation Of Nucleic Acid-Templated Transcription (GO:1903506)	*LIN54;SMAD3;ZNF519;SPIN4;NSD2;HP1BP3;ZNF724;BRCA2;EZH2;SAP30;PHF19*
Negative Regulation Of DNA Recombination (GO:0045910)	*POLQ;PARPBP;KMT5A*
Negative Regulation Of Peptidyl-Threonine Phosphorylation (GO:0010801)	*CALM3;CALM2*
Resolution Of Meiotic Recombination Intermediates (GO:0000712)	*TOP2A;EME1*
Establishment Of Spindle Localization (GO:0051293)	*ESPL1;NUSAP1*
Histone H2A Monoubiquitination (GO:0035518)	*DEPDC1B;BRCA2*
Negative Regulation Of Cilium Assembly (GO:1902018)	*MPHOSPH9;CEP97*
