# Supplementary material for: Decoding Chemotherapy Resistance of Undifferentiated Pleomorphic Sarcoma at the Single Cell Resolution: A Case Report
Source: J Clin Med. 2024 Nov 26;13(23):7176. doi: 10.3390/jcm13237176 (PMC11642494; doi:10.3390/jcm13237176)
Supplement: Supplementary file 1 [file jcm-13-07176-s001.zip › Supplementary Table S3 (track changes).pdf]

Supplementary Table S3. Differentially expressed genes in TME cell types, adjusted p value < 0.05.

Macrophages		T cells		Endothelial cells		COL4A1$^+$ fibroblasts		COL11A1$^+$ fibroblasts		Lymphatic endothelial cells		Mast cells	
Gene	LogFC	Gene	LogFC	Gene	LogFC	Gene	LogFC	Gene	LogFC	Gene	LogFC	Gene	LogFC
CD163	7.32	*CD2*	1.16	*PLVAP*	6.06	*NOTCH3*	5.62	*COL1A2*	3.41	*CCL21*	8.65	*PTGS1*	5.03
CD74	3.71	*TRAC*	8.34	*VWF*	7.25	*CALD1*	3.53	*LUM*	6.41	*FLT4*	5.48	*CPA3*	13.09
MS4A7	6.90	*CD96*	6.13	*PTPRB*	5.90	*COL18A1*	3.90	*CTHRC1*	4.21	*PROX1*	7.67	*HDC*	11.06
MS4A6A	5.47	*CYTIP*	6.83	*NPDC1*	5.07	*CCDC102B*	6.31	*COL11A1*	6.90	*NINL*	5.63	*CLU*	4.58
STAB1	3.33	*IL2RG*	2.84	*FLT1*	6.61	*PDGFRB*	4.02	*COL1A1*	2.84	*SMAD1*	5.24	*VWA5A*	7.61
CSF1R	5.41	*AKNA*	4.09	*DOCK9*	4.43	*COL6A2*	2.66	*COL6A3*	3.13	*TFPI*	4.64	*MS4A2*	10.29
TLR2	6.69	*IKZF1*	2.64	*HSPG2*	4.35	*GJC1*	5.31	*THBS2*	4.33	*TBX1*	7.89	*SLC18A2*	10.09
CD14	5.72	*CORO1A*	2.38	*CLEC14A*	6.84	*UACA*	3.37	*COL3A1*	3.10	*DOCK5*	3.36	*IL1RL1*	8.12
PSAP	3.14	*EVL*	3.17	*PODXL*	5.75	*CACNA1H*	7.02	*SFRP2*	7.79	*ACKR2*	7.79	*GATA2*	7.44
CTSB	3.42	*CXCR4*	1.92	*EGFL7*	3.59	*ARHGEF17*	5.17	*CDH11*	5.17	*STON2*	4.79	*HPGDS*	6.70
C1QA	6.46	*IL32*	2.15	*ADGRL4*	6.18	*PLXDC1*	3.94	*AEBP1*	2.92	*TFF3*	9.08	*HPGD*	7.78
C1QC	5.96	*ETS1*	3.27	*AQP1*	5.08	*ACTA2*	5.49	*DCN*	6.02	*ITGA9*	3.83	*RGS13*	8.20
C1QB	6.27	*NLRC5*	2.01	*MMRN2*	6.00	*COL5A3*	6.92	*COL12A1*	4.36	*STAB2*	7.69	*IL18R1*	6.04
MSR1	6.19	*RESF1*	2.09	*PECAM1*	3.29	*PRKG1*	4.35	*SFRP4*	9.11	*MMRN1*	4.22	*RHEX*	9.29
SLCO2B1	5.13	*ARHGEF1*	1.81	*CDH5*	4.05	*COL6A1*	2.55	*C1S*	4.90	*FAM174B*	5.28	*KIAA1549*	6.56
SAT1	2.64	*BTG1*	1.62	*LIMS2*	5.47	*COL4A2*	2.76	*POSTN*	4.45	*PDPN*	4.78	*KIT*	6.91
FMN1	5.34	*LSP1*	1.76	*EPAS1*	4.33	*TPM1*	2.88	*FBLN1*	5.75	*LAMA4*	3.11	*RAB27B*	6.89
MRC1	4.57	*OGT*	2.08	*RAPGEF5*	4.58	*MYO1B*	3.73	*COL6A1*	2.75	*MGP*	3.24	*TNIK*	4.31
GLUL	3.01	*SYNE2*	1.52	*HECW2*	4.64	*COL4A1*	2.60	*C1R*	5.19	*CLDN5*	4.59	*MLPH*	6.54
DAB2	3.69	*CLEC2D*	2.09	*GRB10*	5.31	*COL6A3*	2.21	*MMP2*	3.18	*KLHL3*	4.61	*CTSG*	11.02
MFSD1	3.47	*WIPF1*	1.88	*ITGA6*	4.51	*HEYL*	6.19	*COMP*	3.36	*EGFL7*	3.56	*TPSG1*	9.02
CYBB	5.44	*RNF213*	1.47	*SHANK3*	5.47	*CD248*	4.53	*ASPN*	5.36	*CFAP54*	5.29	*MCTP2*	4.02
VSIG4	7.02	*ADGRE5*	1.35	*CD34*	4.62	*ADAMTS12*	5.05	*GLI3*	4.49	*RASGRP3*	3.25	*ALOX5*	3.06
TNFSF13	4.85	*TNFAIP3*	1.79	*CALCRL*	4.12	*DLC1*	3.72	*VCAN*	3.73	*CDH5*	2.86	*ATP6V0A2*	3.57
ALOX5	3.48	*ZFP36L2*	1.82	*SLCO2A1*	6.79	*COL3A1*	1.87	*FN1*	2.34	*PPFIBP1*	3.05	*CDK15*	8.29
LYZ	4.07	*TXNIP*	1.36	*COL4A1*	2.96	*COL5A1*	2.16	*CCN2*	3.16	*EFNA5*	6.12	*LIF*	6.34
APOE	5.46	*OGA*	1.47	*SPARCL1*	3.81	*S1PR3*	5.16	*COL6A2*	2.32	*SHC1*	2.89	*CD44*	2.52
F13A1	6.47	*CD44*	1.06	*COL4A2*	2.72	*MCAM*	3.11	*SERPINF1*	3.93	*CALCRL*	2.69	*MT-CO2*	2.58
CSF2RA	3.75	*RABGAP1L*	1.25	*SPRY1*	4.41	*DAAM2*	5.15	*TNC*	5.67	*PIEZO2*	3.92	*SLC45A3*	5.70
GPR34	7.18	*MBNL1*	1.18	*MTUS1*	3.93	*ANO1*	4.21	*COL5A2*	2.57	*SLC38A2*	2.51	*MT-ND4*	2.63
FGD2	4.53	*FNBP1*	1.36	*ARHGAP29*	3.54	*RBPMS*	3.17	*COL5A1*	2.46	*ZNF521*	3.81	*MT-ATP6*	2.89
MAFB	3.24			*ADAMTS9*	4.67	*MYL9*	3.75	*PRRX1*	3.53	*NOVA2*	3.89	*GRAP2*	4.08
CEBPD	2.74			*CDH13*	4.26	*CRISPLD2*	3.81	*KIAA1217*	3.44	*RHOJ*	4.00	*TMEM154*	4.96
FCGR3A	6.11			*INSR*	5.14	*ADAMTS14*	4.65	*CCDC80*	3.55	*ALPK3*	3.67	*MT-ND3*	3.19
FCGR2A	5.62			*APP*	2.40	*PPP1R12B*	3.52	*MRC2*	2.85	*TM4SF1*	3.34	*RGS2*	3.41

MERTK	4.64	*ADGRL2*	5.26	*JAG1*	3.05	*CCN1*	3.29	*TRPC6*	3.35	*FCER1A*	4.98
RGS1	2.36	*VWA1*	5.87	*TAGLN*	3.10	*ITGBL1*	4.65	*BCL6B*	3.97	*ENO2*	4.38
SPI1	4.63	*IGFBP7*	2.23	*TINAGL1*	4.09	*MXRA8*	4.13	*ARHGAP23*	3.51	*TESPA1*	4.86
FOLR2	6.96	*COL15A1*	4.19	*NID2*	2.82	*THBS4*	8.19	*VAV3*	3.88	*RAC2*	3.89
CIITA	2.88	*MCF2L*	4.28	*TPM2*	3.46	*F3*	5.02	*APP*	2.06	*FER*	2.58
RNASE1	3.97	*HYAL2*	3.65	*GGT5*	3.86	*COL14A1*	4.66	*KANK3*	3.65	*PTGS1*	5.03
SLC1A3	4.25	*BCAM*	4.81	*ITGA1*	3.40	*ENPP1*	5.73	*MEGF6*	2.60	*CPA3*	13.09
IFI30	4.92	*PLPP1*	4.01	*THY1*	3.47	*ENAH*	4.08	*DYNC1I2*	1.98	*HDC*	11.06
SGK1	3.26	*PIK3R3*	4.59	*PHLDB1*	2.78	*DLG2*	5.56	*SLC38A1*	2.96	*CLU*	4.58
EMILIN2	4.14	*AFAP1L1*	3.84	*AGRN*	2.92	*PCOLCE*	3.01	*AFDN*	2.74	*VWA5A*	7.61
CD4	2.83	*PTPRM*	3.36	*CSPG4*	3.92	*FSTL1*	2.57	*HYAL2*	2.56	*MS4A2*	10.29
HSPA6	4.26	*ENG*	2.85	*IGFBP7*	2.03	*MEGF6*	3.33	*REXO2*	2.96	*SLC18A2*	10.09
SELENOP	3.96	*DOCK6*	3.68	*PHLDA1*	2.58	*PODN*	3.97	*PPP2R5A*	3.32	*IL1RL1*	8.12
CD68	3.95	*SPARC*	1.54	*NR2F2*	3.20	*ADAM12*	4.52	*KALRN*	2.76	*GATA2*	7.44
PLTP	4.20	*NFIB*	3.52	*NES*	3.13	*COL16A1*	3.24	*FLRT2*	4.24	*HPGDS*	6.70
FCGRT	2.68	*KALRN*	3.61	*PRRX1*	2.86	*NNMT*	3.07	*HSPG2*	1.70	*HPGD*	7.78
CTSZ	2.85	*LAMA5*	3.86	*MFGE8*	2.82	*CTSK*	3.89	*BCAR1*	2.84	*RGS13*	8.20
RASSF4	3.65	*CAV1*	3.49	*ADGRF5*	2.74	*PRKG1*	2.07	*SGIP1*	3.83	*IL18R1*	6.04
FGL2	2.81	*PLPP3*	3.42	*LAMB1*	2.42	*TAGLN*	3.45	*ECSCR*	3.66	*RHEX*	9.29
ADAP2	3.79	*EMP1*	2.81	*SPARC*	1.47	*SETBP1*	2.97	*PGM5*	3.97	*KIAA1549*	6.56
LGMN	3.83	*LAMA4*	2.44	*PCED1A*	2.26	*CNN3*	2.63	*GNG11*	3.21	*KIT*	6.91
TGFBI	2.57	*PTPN14*	3.78	*OLFML2A*	3.18	*CXCL14*	4.65	*AFAP1L1*	3.15	*RAB27B*	6.89
ITGAX	2.98	*COL18A1*	1.52	*NID1*	2.48	*GLT8D2*	4.71	*CELSR1*	3.66	*TNIK*	4.31
MAP3K8	2.84	*DYSF*	4.01	*SPARCL1*	1.89	*CERCAM*	4.02	*PCMTD2*	2.31	*MLPH*	6.54
CPM	2.68	*TM4SF1*	3.75	*LAMB2*	2.55	*CCN5*	4.26	*JUP*	3.39	*CTSG*	11.02
THEMIS2	2.88	*ADGRF5*	2.77	*LAMA4*	1.60	*SMOC2*	4.32	*NRP2*	2.61	*TPSG1*	9.02
PLXDC2	2.42	*TNXB*	3.20	*COL1A2*	1.40	*ECM2*	3.34	*MTUS1*	2.82	*MCTP2*	4.02
ACSL1	3.43	*ADCY4*	3.79	*PCDH1*	2.90	*TPM2*	1.98	*EPHA2*	3.67	*ALOX5*	3.06
FTH1	2.44	*PRSS23*	2.98	*COL16A1*	2.43	*FAP*	3.35	*FHL3*	2.96	*ATP6V0A2*	3.57
LHFPL2	3.29	*SPTBN1*	3.03	*LPP*	1.88	*LOXL1*	3.18	*DOCK9*	1.94	*CDK15*	8.29
GPNMB	3.23	*SELENOW*	2.86	*OSMR*	2.65	*FBN1*	2.15	*ELK3*	3.01	*LIF*	6.34
CST3	1.74	*CRIP2*	3.55	*PCOLCE*	2.28	*ADAMTSL1*	3.30	*PRKAR1B*	3.83	*CD44*	2.52
NPC2	2.92	*PTPRK*	2.99	*VMP1*	1.47	*SULF1*	3.21	*PPM1F*	2.57	*MT-CO2*	2.58
GPR183	2.63	*CD93*	2.22	*SMTN*	2.59	*COL8A1*	2.77	*DIPK2B*	3.18	*SLC45A3*	5.70
SRGN	1.64	*ADAM15*	2.97	*TIMP3*	2.10	*ANTXR1*	2.74	*HECW2*	2.15	*MT-ND4*	2.63
GRN	2.28	*MCAM*	2.75	*TNS1*	2.37	*THBS3*	2.77	*PPP1R13B*	3.76	*MT-ATP6*	2.89
KCTD12	2.52	*S1PR1*	3.31	*ARHGEF7*	2.11	*FGFR1*	2.26	*ARHGAP29*	2.53	*GRAP2*	4.08
HERPUD1	2.00	*IVNS1ABP*	2.60	*COL5A2*	1.51	*DNM1*	3.48	*HSPB1*	2.41	*TMEM154*	4.96
GRINA	2.71	*BHLHE40*	2.57	*ST5*	2.27	*BNC2*	2.94	*CD200*	3.77	*MT-ND3*	3.19
TYMP	2.35	*PLXND1*	1.71	*COL1A1*	1.04	*FRMD6*	3.97	*RBPMS*	2.48	*RGS2*	3.41

TNFAIP2	2.49	*GSN*	1.59	*HIPK2*	2.05	*LRRC15*	3.30	*UNC5B*	3.17	*FCER1A*	4.98
AP2A2	2.58	*NEDD9*	2.44	*MICALL2*	2.22	*LAMB1*	1.45	*CAVIN2*	2.98	*ENO2*	4.38
DSE	2.58	*ECE1*	2.80	*FBN1*	1.44	*CALD1*	1.50	*ERICH1*	2.22	*TESPA1*	4.86
CTSS	2.70	*ENTPD1*	2.38	*UTRN*	1.60	*LRP1*	1.75	*TMOD2*	3.39	*RAC2*	3.89
RGL1	2.49	*LMNA*	1.37	*PXDN*	1.45	*FKBP10*	2.60	*SH2D3C*	2.87	*FER*	2.58
DPYD	2.06	*CEMIP2*	2.54	*LOXL2*	1.98	*CFH*	3.66	*ROBO4*	3.24		
FCHO2	2.19	*ITGA5*	2.36	*FHOD1*	2.03	*PLAGL1*	3.25	*GRPEL2*	3.61		
LAPTM5	2.00	*ETS1*	1.19	*SLC38A2*	1.73	*EMILIN1*	3.10	*PXDN*	2.57		
GAA	2.33	*CD9*	1.81	*MAT2A*	1.30	*EGR1*	2.15	*RIMKLB*	2.85		
FKBP5	1.53	*TMEM255B*	2.34	*IGFBP4*	1.47	*FAT1*	2.56	*TP53I3*	2.90		
SMAP2	1.78	*PALM2-AKAP2*	2.30	*MAP4*	1.24	*NBL1*	2.79	*CCDC102B*	1.01		
ITPR2	2.09	*LAMB1*	1.91	*PDLIM7*	1.63	*MXRA5*	1.66	*MYO18A*	2.43		
HCLS1	1.74	*SERPINH1*	1.84	*BGN*	1.12	*THBS1*	2.77	*MYH10*	3.45		
ZEB2	1.98	*RASGRP3*	2.08	*FNDC3B*	1.10	*OLFML3*	2.97	*PTPRE*	1.86		
HAVCR2	1.48	*KLF2*	1.70	*ASAP1*	1.33	*BGN*	1.72	*IGF1*	3.44		
PLD3	2.38	*AGRN*	1.78			*SPON2*	3.48	*NARF*	2.33		
GNA13	2.03	*HDAC7*	2.08			*OSMR*	2.42	*TSPAN18*	3.42		
TPP1	1.92	*DOCK1*	2.06			*MGP*	2.04	*TMEM255B*	2.28		
FGD4	2.13	*CNN3*	1.45			*SERPINE1*	2.39	*TIE1*	3.12		
RNF149	1.68	*RGCC*	2.15			*SPARC*	1.54	*CNKSR3*	3.36		
REL	2.07	*YBX3*	1.94			*PTPRG*	2.09	*IGFBP7*	1.68		
IFNGR1	2.20	*LOXL2*	2.09			*MYL9*	2.03	*SPTBN1*	2.04		
LRP1	1.71	*SHC1*	1.89			*TPM1*	1.87	*CHST15*	3.17		
ARHGAP18	1.72	*PHLDB1*	1.48			*LIMA1*	2.43	*EMP1*	1.91		
METRNL	1.79	*SEC14L1*	1.78			*GJA1*	2.81	*PILRB*	1.53		
MCL1	1.00	*TCF4*	2.00			*NFKBIZ*	1.54	*UHRF2*	2.39		
RIN3	1.80	*MYOF*	1.74			*ADAMTS2*	2.24	*PRSS23*	2.19		
DOCK2	1.29	*SLC2A3*	2.02			*CRISPLD2*	2.40	*ASAP1*	1.39		
ARAP1	1.78	*NOTCH1*	1.82			*ISLR*	2.77	*BICD1*	2.49		
SLA	1.32	*STOM*	1.85			*LTBP3*	1.75	*HOMER3*	2.26		
ZFP36	1.25	*NAV1*	1.73			*INHBA*	2.54	*EPB41L2*	1.88		
ARHGAP4	1.24	*ARGLU1*	1.02			*MMP14*	1.66	*TCF4*	2.47		
GNAQ	1.44	*TGFBR2*	1.25			*PDLIM7*	2.34	*CLU*	2.26		
CYBA	1.48	*NRP1*	1.29			*HTRA1*	1.77	*HSP90B1*	1.84		
CNDP2	1.73	*CCN1*	1.53			*PHLDB1*	1.76	*TMCO3*	2.42		
DOCK4	1.46	*CNST*	1.80			*PLXDC1*	1.40	*HDAC7*	1.85		
QKI	1.22	*A2M*	1.30			*PDGFRB*	1.72	*PRKG1*	2.04		
CHST11	1.14	*TACC1*	1.34			*CXCL12*	2.24	*KANK2*	2.77		
SFMBT2	1.25	*PXN*	1.51			*ANGPTL2*	2.25	*UBA6*	2.05		

PER1	1.04
CD63	1.04
FOS	1.03
MARCKS	1.16

MYO15B	1.38
SYNE2	1.20
ACTN4	1.45
ZBTB16	1.00
FSTL1	1.02
IGFBP4	1.52
INF2	1.36
FBN1	1.18
TIMP3	1.09
CTNND1	1.17
DDIT4	1.18
MSN	1.05
PTMS	1.05

LTBP2	2.08
PPFIBP1	2.01
GOLM1	2.57
THY1	1.91
SERPINH1	1.79
TIMP2	1.31
RCN3	1.84
DPYSL3	1.82
PLAU	2.51
TMEM263	2.17
DPT	1.87
DLC1	1.56
SEPTIN11	2.21
CALU	1.65
FOXO1	2.04
TIMP3	1.76
ELN	1.92
RORA	1.75
COL15A1	1.11
SULF2	1.83
TTC3	1.39
CEMIP	2.05
VMP1	1.08
EMP1	1.08
LAMB2	1.55
FAM3C	2.09
ANXA5	1.30
ZNF532	1.86
OLFML2B	1.78
GALNS	1.61
BCAR1	1.73
ITGAV	1.44
RRBP1	1.22
CCNL2	1.09
COL1A2	3.41

PHLDB2	2.54
NCKAP1	2.84
NOD1	2.63
SVIL	2.57
TXNDC5	2.84
DHX15	1.40
PTPRM	1.68
OAF	2.89
PDE4D	2.18
ARHGAP17	2.28
NFAT5	2.08
NID1	1.70
EMILIN1	2.24
LAMC1	2.26
CCNL2	1.08
FAM107B	2.87
DYSF	2.33
ADAM10	1.92
MED10	2.12
TJP1	2.06
LENG8	1.17
NFIB	2.25
DOCK6	2.07
TRIOBP	1.92
ITGA5	1.42
MYO6	2.65
DCTN1	1.50
CAVIN1	2.00
SFPQ	1.83
S100A16	2.59
DUSP6	2.41
LMBR1	2.18
ATG4B	1.23
RAPGEF5	2.26
REEP3	2.33
CFI	2.44
PLCG1	1.92
OSBPL9	1.79
