# Supplementary material for: Decoding Chemotherapy Resistance of Undifferentiated Pleomorphic Sarcoma at the Single Cell Resolution: A Case Report
Source: J Clin Med. 2024 Nov 26;13(23):7176. doi: 10.3390/jcm13237176 (PMC11642494; doi:10.3390/jcm13237176)
Supplement: Supplementary file 1 [file jcm-13-07176-s001.zip › Supplementary Table S4.pdf]

Supplementary Table S4. Signaling pathways (KEGG) and biological processes (GO) enriched in TME subpopulations, adjusted p value < 0.05.

Macrophages			
KEGG Term	Genes	GO Term	Genes
Lysosome	*CD63;NPC2;GAA;PSAP;CTSZ;LAPTM5;TPP1;CD68;CTSS;LGMN;CTSB*	Astrocyte Activation (GO:0048143)	*C1QA;GRN;LRP1;IFNGR1*
Antigen processing and presentation	*CD74;CIITA;CD4;HSPA6;IFI30;CTSS;CTSB;LGMN*	Receptor-Mediated Endocytosis (GO:0006898)	*MSR1;DAB2;LRP1;STAB1;MRC1;FCHO2;APOE;AP2A2*
Tuberculosis	*CD74;CIITA;FCGR3A;FCGR2A;IFNGR1;MRC1;ITGAX;CD14;CTSS;TLR2*	Inflammatory Response (GO:0006954)	*CSF1R;CIITA;REL;CYBB;CYBA;CD14;FOS;LYZ;FOLR2;TLR2*
Phagosome	*MSR1;FCGR3A;FCGR2A;MRC1;CYBB;CYBA;CD14;CTSS;TLR2*	Endocytosis (GO:0006897)	*MSR1;DAB2;LRP1;STAB1;MRC1;RIN3;CD14;APOE;MERTK*
Leishmaniasis	*FCGR3A;FCGR2A;IFNGR1;CYBB;CYBA;FOS;TLR2*	Glycolipid Transport (GO:0046836)	*NPC2;PSAP;PLTP*
Chagas disease	*C1QB;C1QA;IFNGR1;GNAQ;FOS;TLR2;C1QC*	Negative Regulation Of Gene Expression (GO:0010629)	*SRGN;MSR1;ZFP36;SPI1;LRP1;GPNMB;SFMBT2;REL;APOE;LGMN;HAVCR2*
Osteoclast differentiation	*CSF1R;FCGR3A;SPI1;FCGR2A;IFNGR1;CYBA;FOS*	Negative Regulation Of Interleukin-2 Production (GO:0032703)	*ZFP36;LAPTM5;VSIG4;HAVCR2*
Complement and coagulation cascades	*C1QB;C1QA;ITGAX;F13A1;VSIG4;C1QC*	Positive Regulation Of Tumor Necrosis Factor Production (GO:0032760)	*FCGR3A;IFNGR1;CYBA;CD14;HAVCR2;TLR2*
Coronavirus disease	*C1QB;C1QA;FCGR2A;F13A1;CYBB;FOS;TLR2;C1QC*	Positive Regulation Of Endocytosis (GO:0045807)	*DAB2;LRP1;CYBA;APOE;DOCK2;MERTK*
Pertussis	*C1QB;C1QA;FOS;CD14;C1QC*	Regulation Of Interleukin-2 Production (GO:0032663)	*ZFP36;CD4;LAPTM5;VSIG4;HAVCR2*
Ferroptosis	*ACSL1;FTH1;CYBB;SAT1*	Positive Regulation Of Tumor Necrosis Factor Superfamily Cytokine Production (GO:1903557)	*FCGR3A;IFNGR1;CYBA;CD14;HAVCR2;TLR2*

Apoptosis	*CTSZ;ITPR2;FOS;CTSS;MCL1;CTSB*	Regulation Of Tumor Necrosis Factor Production (GO:0032680)	*ZFP36;FCGR3A;IFNGR1;CYBA;CD14;HAVCR2;TLR2*
Cholesterol metabolism	*LRP1;NPC2;APOE;PLTP*	Negative Regulation Of Macromolecule Metabolic Process (GO:0010605)	*MSR1;SPI1;LRP1;SFMBT2;REL;APOE;LGMN;HAVCR2*
Staphylococcus aureus infection	*C1QB;C1QA;FCGR3A;FCGR2A;C1QC*	Protein Catabolic Process (GO:0030163)	*CTSZ;TPP1;APOE;CTSS;LGMN;CTSB*
Parathyroid hormone synthesis, secretion and action	*GNA13;MAFB;GNAQ;ITPR2;FOS*	Regulation Of Intracellular Signal Transduction (GO:1902531)	*GNA13;CSF1R;FGD4;SLA;ARHGAP18;ARAP1;SGK1;DOCK2;ARHGAP4*
Toxoplasmosis	*CIITA;IFNGR1;ALOX5;HSPA6;TLR2*	Regulation Of Small GTPase Mediated Signal Transduction (GO:0051056)	*GNA13;FGD4;ARHGAP18;ARAP1;DOCK2;ARHGAP4*
Prion disease	*C1QB;C1QA;HSPA6;CYBB;ITPR2;CYBA;C1QC*	Regulation Of Amyloid-Beta Clearance (GO:1900221)	*LRP1;IFNGR1;APOE*
Systemic lupus erythematosus	*C1QB;C1QA;FCGR3A;FCGR2A;C1QC*	Response To Cytokine (GO:0034097)	*CSF1R;CD74;CIITA;REL;HCLS1;MCL1*
Estrogen signaling pathway	*GNAQ;HSPA6;ITPR2;FOS;FKBP5*	Negative Regulation Of T Cell Activation (GO:0050868)	*GPNMB;LAPTM5;VSIG4;HAVCR2*
Lipid and atherosclerosis	*HSPA6;CYBB;CYBA;CD14;FOS;TLR2*	Microglial Cell Activation (GO:0001774)	*C1QA;GRN;IFNGR1*
PD-L1 expression and PD-1 checkpoint pathway in cancer	*CD4;IFNGR1;FOS;TLR2*	Positive Regulation Of Transport (GO:0051050)	*DAB2;LRP1;APOE;SGK1*
Salivary secretion	*CST3;GNAQ;ITPR2;LYZ*	Regulation Of Angiogenesis (GO:0045765)	*GRN;GPNMB;ALOX5;STAB1;ITGAX;EMILIN2;GLUL*
Hematopoietic cell lineage	*CSF1R;CD4;CD14;CSF2RA*	Positive Regulation Of Cholesterol Efflux (GO:0010875)	*LRP1;APOE;PLTP*
Toll-like receptor signaling pathway	*MAP3K8;CD14;FOS;TLR2*	Negative Regulation Of Erythrocyte Differentiation (GO:0045647)	*ZFP36;MAFB*

Endocytosis	*DAB2;HSPA6;ARAP1;FOLR2;SMAP2;AP2A2*	Cell Junction Disassembly (GO:0150146)	*C1QB;C1QC*
Legionellosis	*HSPA6;CD14;TLR2*	Humoral Immune Response Mediated By Circulating Immunoglobulin (GO:0002455)	*C1QB;C1QC*
Glutamatergic synapse	*GNAQ;ITPR2;SLC1A3;GLUL*	Regulation Of Phagocytosis (GO:0050764)	*CYBA;DOCK2;MERTK;TLR2*
Neutrophil extracellular trap formation	*FCGR3A;FCGR2A;CYBB;CYBA;TLR2*	Lipid Transport (GO:0006869)	*ACSL1;NPC2;SLCO2B1;APOE;PLTP*
Long-term depression	*GNA13;GNAQ;ITPR2*	Antigen Processing And Presentation Of Exogenous Peptide Antigen Via MHC Class II (GO:0019886)	*IFI30;CTSS;LGMN*
Platelet activation	*GNA13;FCGR2A;GNAQ;ITPR2*	Regulation Of Digestive System Process (GO:0044058)	*SGK1;TYMP*
Acute myeloid leukemia	*CSF1R;SPI1;CD14*	Positive Regulation Of Amyloid-Beta Clearance (GO:1900223)	*LRP1;APOE*
Renin secretion	*GNAQ;ITPR2;CTSB*	Positive Regulation Of Receptor Catabolic Process (GO:2000646)	*LAPTM5;APOE*
Human immunodeficiency virus 1 infection	*CD4;GNAQ;ITPR2;FOS;TLR2*	Regulation Of Peptidase Activity (GO:0052547)	*CST3;CTSS;CTSB*
Yersinia infection	*CD4;FCGR2A;GNAQ;FOS*	Antigen Processing And Presentation Of Peptide Antigen Via MHC Class II (GO:0002495)	*IFI30;CTSS;LGMN*
		Positive Regulation Of Cytokine Production (GO:0001819)	*CSF1R;CD74;CD4;LAPTM5;CYBA;CD14;HAVCR2;TLR2*
		Integrated Stress Response Signaling (GO:0140467)	*MAFB;CEBPD;FOS*
		Negative Regulation Of Cytokine Production (GO:0001818)	*SRGN;ZFP36;GPNMB;LAPTM5;VSIG4;HAVCR2*

Positive Regulation Of ERK1 And ERK2 Cascade (GO:0070374)	*CSF1R;CD74;CD4;GPNMB;GPR183;APOE*
Phagocytosis (GO:0006909)	*MSR1;LRP1;CD14;MERTK*
Proteolysis (GO:0006508)	*SRGN;CPM;CTSZ;TPP1;CTSS;CNDP2;LGMN;CTSB*
Antigen Processing And Presentation Of Exogenous Peptide Antigen (GO:0002478)	*IFI30;CTSS;LGMN*
Positive Regulation Of Cholesterol Transport (GO:0032376)	*LRP1;APOE;PLTP*
Myeloid Leukocyte Differentiation (GO:0002573)	*CSF1R;CD4;SPI1;GPR183*
Regulation Of Cholesterol Efflux (GO:0010874)	*LRP1;APOE;PLTP*
Amide Transport (GO:0042886)	*SLC1A3;FOLR2;PLTP*
Regulation Of Defense Response To Bacterium (GO:1900424)	*GRN;EMILIN2*
Synapse Pruning (GO:0098883)	*C1QB;C1QC*
Regulation Of Cell Migration (GO:0030334)	*CSF1R;CD74;DAB2;GRN;GPNMB;ITGAX;EMILIN2;SGK1;ARHGAP4*
Macrophage Activation (GO:0042116)	*C1QA;FCGR3A;IFNGR1*
Proteolysis Involved In Protein Catabolic Process (GO:0051603)	*CTSZ;CTSS;CTSB;LGMN*
Regulation Of Granulocyte Differentiation (GO:0030852)	*HCLS1;C1QC*
Positive Regulation Of Monocyte Differentiation (GO:0045657)	*CD74;CD4*
Regulation Of Erythrocyte Differentiation (GO:0045646)	*ZFP36;SPI1;MAFB*

Lytic Vacuole Organization (GO:0080171)	*GRN;GAA;TPP1*
Positive Regulation Of Defense Response To Bacterium (GO:1900426)	*GRN;EMILIN2*
Positive Regulation Of Pattern Recognition Receptor Signaling Pathway (GO:0062208)	*CYBA;CD14;TLR2*
Positive Regulation Of Transferase Activity (GO:0051347)	*CSF1R;CD74;CD4;APOE;MERTK*
Regulation Of Kinase Activity (GO:0043549)	*CSF1R;CD74;CD4;MERTK*
Positive Regulation Of Myeloid Leukocyte Differentiation (GO:0002763)	*CD74;CD4;HCLS1*
Regulation Of Chemokine Production (GO:0032642)	*CSF1R;CD74;TLR2*
Positive Regulation Of Response To Biotic Stimulus (GO:0002833)	*GRN;EMILIN2;CYBA*
Negative Regulation Of Neuron Death (GO:1901215)	*GRN;GPNMB;APOE;LGMN*
Negative Regulation Of Leukocyte Apoptotic Process (GO:2000107)	*HCLS1;MERTK*
Cellular Response To Thyroid Hormone Stimulus (GO:0097067)	*CTSS;CTSB*
Positive Regulation Of Lipid Transport (GO:0032370)	*LRP1;APOE*
Lysosome Organization (GO:0007040)	*GRN;GAA;TPP1*

Defense Response To Tumor Cell (GO:0002357)	*SPI1;LAPTM5*
Response To Thyroid Hormone (GO:0097066)	*CTSS;CTSB*
Regulation Of Viral Entry Into Host Cell (GO:0046596)	*CD74;CIITA;CD4*
Negative Regulation Of Apoptotic Process (GO:0043066)	*CD74;DAB2;GRN;HCLS1;APOE;MERTK;MCL1;LGMN;GRINA*
Positive Regulation Of Cell Population Proliferation (GO:0008284)	*CSF1R;CD74;GRN;TNFSF13;ITGAX;HCLS1;FOLR2;CSF2RA;LGMN*
Positive Regulation Of MAPK Cascade (GO:0043410)	*CSF1R;CD74;CD4;GPNMB;GPR183;LAPTM5;APOE*
Positive Regulation Of Phosphorylation (GO:0042327)	*CSF1R;CD74;DAB2;CD4;GPNMB;MERTK*
Regulation Of Cytokine Production Involved In Inflammatory Response (GO:1900015)	*PER1;ALOX5;PLD3*
Vacuolar Transport (GO:0007034)	*GRN;LRP1;PSAP*
Regulation Of Gene Expression (GO:0010468)	*MSR1;CD74;SPI1;LRP1;IFNGR1;SFMBT2;PER1;MAFB;REL;ITGAX;HCLS1;APOE;LGMN;HAVCR2;TLR2*
Regulation Of Extracellular Matrix Disassembly (GO:0010715)	*CST3;LRP1*
Ceramide Transport (GO:0035627)	*PSAP;PLTP*
Regulation Of Cytokine Production (GO:0001817)	*SRGN;PER1;GPNMB;ALOX5;PLD3*

		Regulation Of ERK1 And ERK2 Cascade (GO:0070372)	*CSF1R;CD74;CD4;GPNMB;GPR183;APOE*
		Positive Regulation Of Kinase Activity (GO:0033674)	*CSF1R;CD74;CD4;MERTK*
		Cholesterol Transport (GO:0030301)	*MSR1;NPC2;APOE*
		Negative Regulation Of Immune Effector Process (GO:0002698)	*GRN;HAVCR2*
		Regulation Of Monocyte Differentiation (GO:0045655)	*CD74;CD4*
		Astrocyte Development (GO:0014002)	*C1QA;IFNGR1*
		High-Density Lipoprotein Particle Remodeling (GO:0034375)	*APOE;PLTP*
		Lysosomal Transport (GO:0007041)	*GRN;LRP1;PSAP;LAPTM5*
		Positive Regulation Of B Cell Activation (GO:0050871)	*CD74;SPI1;GPR183*
T cells			
Endothelial cells			
Focal adhesion	*LAMA5;FLT1;TNXB;VWF;SHC1;CAV1;LAMA4;PXN;PIK3R3;LAMB1;ACTN4;COL4A2;COL4A1;ITGA6;ITGA5;DOCK1*	Regulation Of Cell Migration (GO:0030334)	*NOTCH1;FLT1;TNXB;PLXND1;INSR;CAV1;PIK3R3;NEDD9;LAMB1;ACTN4;PTPRK;SYNE2;CDH5;ADAM15;PODXL;MMRN2;LMNA;PECAM1;CDH13;ITGA6;ENG*
ECM-receptor interaction	*LAMA5;TNXB;VWF;COL4A2;COL4A1;LAMA4;ITGA6;LAMB1;ITGA5;AGRN;HSPG2*	Positive Regulation Of Cell Motility (GO:2000147)	*NOTCH1;FLT1;INSR;CAV1;PIK3R3;NEDD9;LAMB1;ACTN4;SYNE2;CDH5;PODXL;PECAM1;CDH13;ITGA6*
PI3K-Akt signaling pathway	*LAMA5;FLT1;TNXB;VWF;INSR;LAMA4;PIK3R3;LAMB1;COL4*	Positive Regulation Of Cell Migration (GO:0030335)	*NOTCH1;FLT1;INSR;CAV1;PIK3R3;NEDD9;LAMB1;ACTN4;SYNE2;CD*

	A2;COL4A1;DDIT4;ITGA6;ITGA5		*H5;PODXL;PECAM1;CDH13;ITGA6;DOCK1*
Human papillomavirus infection	*LAMA5;NOTCH1;TNXB;COL4A2;VWF;COL4A1;LAMA4;PXN;PIK3R3;ITGA6;LAMB1;ITGA5*	Regulation Of Angiogenesis (GO:0045765)	*CDH5;SPARC;RGCC;FLT1;PLXND1;COL4A2;CEMIP2;PTPRM;ETS1;HSPG2;CD34;AQP1*
Small cell lung cancer	*LAMA5;COL4A2;COL4A1;LAMA4;PIK3R3;ITGA6;LAMB1*	Negative Regulation Of Endothelial Cell Proliferation (GO:0001937)	*FLT1;SPARC;RGCC;CAV1;MMRN2;PTPRM*
Amoebiasis	*LAMA5;COL4A2;COL4A1;LAMA4;PIK3R3;LAMB1;ACTN4*	Extracellular Matrix Organization (GO:0030198)	*APP;COL18A1;COL15A1;GSN;COL4A2;ADAM15;COL4A1;SERPINH1;ADAMTS9;LOXL2*
Adherens junction	*PTPRB;INSR;CTNND1;PTPRM;ACTN4;TGFBR2*	Endothelial Cell Migration (GO:0043542)	*CDH5;PLXND1;PXN;CDH13;FSTL1;LOXL2*
Pathways in cancer	*LAMA5;NOTCH1;EPAS1;ZBTB16;LAMA4;ADCY4;PIK3R3;LAMB1;ETS1;RASGRP3;TGFBR2;COL4A2;COL4A1;ITGA6*	Negative Regulation Of Cellular Process (GO:0048523)	*APP;COL18A1;NOTCH1;PLXND1;PTPRK;ETS1;PTPN14;RGCC;ADAM15;PODXL;HYAL2;MMRN2;LMNA;CDH13;IGFBP7;PLPP1*
Leukocyte transendothelial migration	*CDH5;CTNND1;PXN;PECAM1;PIK3R3;MSN;ACTN4*	Blood Vessel Morphogenesis (GO:0048514)	*EGFL7;FLT1;COL4A1;CAV1;TGFBR2;ENG*
Bacterial invasion of epithelial cells	*SHC1;CAV1;PXN;PIK3R3;ITGA5;DOCK1*	Integrin-Mediated Signaling Pathway (GO:0007229)	*LAMA5;ADAM15;NEDD9;ITGA6;ITGA5;PLPP3;DOCK1*
Regulation of actin cytoskeleton	*GSN;PXN;PIK3R3;MSN;ITGA6;ACTN4;ITGA5;DOCK1*	Negative Regulation Of Angiogenesis (GO:0016525)	*SPARC;RGCC;COL4A2;PTPRM;ADAMTS9;HSPG2;KLF2*
Relaxin signaling pathway	*COL4A2;COL4A1;SHC1;ADCY4;PIK3R3;TGFBR2*	Vasculogenesis (GO:0001570)	*EGFL7;NOTCH1;CAV1;TGFBR2;ENG*
Proteoglycans in cancer	*CAV1;PXN;PIK3R3;MSN;TIMP3;ITGA5;HSPG2*	Negative Regulation Of Anoikis (GO:2000811)	*NOTCH1;CAV1;PIK3R3;ITGA5*
Phospholipase D signaling pathway	*SHC1;INSR;ADCY4;PIK3R3;PLPP3;PLPP1*	Regulation Of Protein Tyrosine Kinase Activity (GO:0061097)	*APP;SHC1;HYAL2;CAV1;NEDD9*
Fc gamma R-mediated phagocytosis	*GSN;PIK3R3;PLPP3;DOCK1;PLPP1*	Negative Regulation Of Cell Population Proliferation (GO:0008285)	*APP;COL18A1;NOTCH1;RGCC;MMRN2;LMNA;CDH13;IGFBP7;PTPRK;ETS1;PTPN14;PLPP1*

Rap1 signaling pathway	*FLT1;INSR;CTNND1;ADCY4;PIK3R3;RAPGEF5;RASGRP3*	Negative Regulation Of Epithelial Cell Proliferation (GO:0050680)	*SPARC;RGCC;NFIB;CAV1;PTPRM;PTPRK*
Ras signaling pathway	*FLT1;SHC1;INSR;PIK3R3;RAPGEF5;ETS1;RASGRP3*	Regulation Of Cell Population Proliferation (GO:0042127)	*APP;COL18A1;NOTCH1;TNXB;SHC1;INSR;PTPRK;ETS1;PTPN14;TGFBR2;RGCC;MMRN2;LMNA;CDH13;IGFBP7;PLPP1;ENG*
FoxO signaling pathway	*INSR;S1PR1;PIK3R3;KLF2;TGFBR2*	Regulation Of Anoikis (GO:2000209)	*NOTCH1;CAV1;PIK3R3;ITGA5*
Fluid shear stress and atherosclerosis	*CDH5;CAV1;PECAM1;PIK3R3;KLF2*	Positive Regulation Of Cardiac Epithelial To Mesenchymal Transition (GO:0062043)	*NOTCH1;ENG;TGFBR2*
Cell adhesion molecules	*CDH5;PECAM1;PTPRM;ITGA6;CD34*	Epithelial Cell Migration (GO:0010631)	*PLXND1;PXN;CDH13;FSTL1;LOXL2*
Dilated cardiomyopathy	*LMNA;ADCY4;ITGA6;ITGA5*	Positive Regulation Of Epithelial To Mesenchymal Transition (GO:0010718)	*NOTCH1;TNXB;RGCC;LOXL2;TGFBR2*
Hematopoietic cell lineage	*CD9;ITGA6;ITGA5;CD34*	Central Nervous System Development (GO:0007417)	*APP;GSN;COL4A1;NFIB;ZBTB16;DDIT4;HSPG2;SPTBN1;ADGRL2;TGFBR2*
MicroRNAs in cancer	*NOTCH1;TNXB;SHC1;DDIT4;PIK3R3;TIMP3;ITGA5*	Transmembrane Receptor Protein Tyrosine Kinase Signaling Pathway (GO:0007169)	*FLT1;COL4A2;SHC1;INSR;DDIT4;PXN;GRB10;PIK3R3;NEDD9;KALRN*
AGE-RAGE signaling pathway in diabetic complications	*COL4A2;COL4A1;PIK3R3;TGFBR2*	Regulation Of Epithelial To Mesenchymal Transition (GO:0010717)	*PHLDB1;TNXB;RGCC;SPRY1;LOXL2;TGFBR2*
Protein digestion and absorption	*COL18A1;COL15A1;COL4A2;COL4A1*	Regulation Of Anatomical Structure Morphogenesis (GO:0022603)	*PHLDB1;SPARC;INF2;PLXND1;HECW2;ETS1;SHANK3*
Regulation of lipolysis in adipocytes	*INSR;ADCY4;PIK3R3*	Positive Regulation Of Macromolecule Metabolic Process (GO:0010604)	*APP;CDH5;NOTCH1;RGCC;GSN;PIK3R3;MSN;ECE1;ETS1;CD34;KLF2*

Toxoplasmosis	*LAMA5;LAMA4;ITGA6;LAMB1*	Regulation Of Endothelial Cell Proliferation (GO:0001936)	*EGFL7;SPARC;RGCC;CAV1;PTPRM;CDH13*
		Cell Junction Assembly (GO:0034329)	*CDH5;PLXND1;CTNND1;CDH13;ITGA6;SHANK3*
		Cellular Response To Transforming Growth Factor Beta Stimulus (GO:0071560)	*CDH5;HYAL2;PXN;PTPRK;TGFBR2;ENG*
		Endothelium Development (GO:0003158)	*PECAM1;CD34;FSTL1*
		Cell-Matrix Adhesion (GO:0007160)	*ADAM15;ITGA6;PTPRK;ITGA5;ADAMTS9;CD34*
		Negative Regulation Of Endothelial Cell Migration (GO:0010596)	*NOTCH1;RGCC;PTPRM;ADAMTS9*
		Cellular Response To Hypoxia (GO:0071456)	*NOTCH1;RGCC;EPAS1;LMNA;AQP1*
		Transforming Growth Factor Beta Receptor Signaling Pathway (GO:0007179)	*CDH5;PXN;PTPRK;TGFBR2;ENG*
		Cellular Response To Decreased Oxygen Levels (GO:0036294)	*NOTCH1;RGCC;EPAS1;LMNA;AQP1*
		Brain Development (GO:0007420)	*COL4A1;NFIB;DDIT4;HSPG2;SHANK3;ADGRL2;TGFBR2*
		Positive Regulation Of Angiogenesis (GO:0045766)	*CDH5;FLT1;ITGA5;ETS1;CD34;AQP1*
		Negative Regulation Of Blood Vessel Morphogenesis (GO:2000181)	*SPARC;RGCC;COL4A2;PTPRM;HSPG2*
		Aorta Morphogenesis (GO:0035909)	*ADAMTS9;TGFBR2;ENG*
		Heart Trabecula Morphogenesis (GO:0061384)	*NOTCH1;S1PR1;ENG*

Circulatory System Development (GO:0072359)	*CDH5;NOTCH1;ECE1;HSPG2;FBN1;TGFBR2*
Insulin Receptor Signaling Pathway (GO:0008286)	*SHC1;INSR;GRB10;PIK3R3*
Cardiac Epithelial To Mesenchymal Transition (GO:0060317)	*NOTCH1;SPRY1;ENG*
Regulation Of Endothelial Cell Migration (GO:0010594)	*SPARC;PTPRM;PLPP3;ETS1;ADAMTS9*
Positive Regulation Of Signal Transduction (GO:0009967)	*TNXB;VWF;GRB10;LAMB1;ITGA5;SHANK3;KLF2;LIMS2*
Endocardial Cushion Morphogenesis (GO:0003203)	*NOTCH1;TGFBR2;ENG*
Regulation Of Substrate Adhesion-Dependent Cell Spreading (GO:1900024)	*NEDD9;ACTN4;DOCK1;LIMS2*
Sprouting Angiogenesis (GO:0002040)	*CDH13;PIK3R3;LOXL2;ENG*
Positive Regulation Of Transmembrane Receptor Protein Serine/Threonine Kinase Signaling Pathway (GO:0090100)	*CDH5;NOTCH1;TNXB;TGFBR2;ENG*
Regulation Of Vascular Endothelial Growth Factor Receptor Signaling Pathway (GO:0030947)	*MMRN2;GRB10;ITGA5*
Glomerulus Vasculature Development (GO:0072012)	*PECAM1;CD34*
Venous Blood Vessel Morphogenesis (GO:0048845)	*NOTCH1;ENG*
Regulation Of GTPase Activity (GO:0043087)	*DOCK6;PLXND1;DOCK9;ITGA6;RAPGEF5;AGRN;RASGRP3*

Positive Regulation Of Cell Differentiation (GO:0045597)	*RGCC;TNXB;ZBTB16;TCF4;LAMB1;CD34;LOXL2;TGFBR2*
Positive Regulation Of BMP Signaling Pathway (GO:0030513)	*CDH5;NOTCH1;ENG*
Positive Regulation Of Vasculature Development (GO:1904018)	*CDH5;FLT1;ETS1;CD34;AQP1*
Cell-Cell Adhesion Mediated By Cadherin (GO:0044331)	*CDH5;CTNND1;CDH13*
Regulation Of Extracellular Matrix Assembly (GO:1901201)	*NOTCH1;RGCC*
Cellular Hyperosmotic Response (GO:0071474)	*YBX3;AQP1*
Vascular Associated Smooth Muscle Cell Development (GO:0097084)	*NOTCH1;ENG*
Vascular Wound Healing (GO:0061042)	*MCAM;CD34*
Regulation Of Cell Adhesion Molecule Production (GO:0060353)	*NOTCH1;CAV1*
Negative Regulation Of Cell Migration (GO:0030336)	*ADAM15;HYAL2;MMRN2;NEDD9;PTPRK;ENG*
Extracellular Structure Organization (GO:0043062)	*APP;COL15A1;COL4A2;COL4A1;ADAMTS9*
Adenylate Cyclase-Activating G Protein-Coupled Receptor Signaling Pathway (GO:0007189)	*CALCRL;ADGRF5;ADCY4;ADGRL4;ADGRL2*
External Encapsulating Structure Organization (GO:0045229)	*APP;COL15A1;COL4A2;COL4A1;ADAMTS9*

Positive Regulation Of Protein Phosphorylation (GO:0001934)	*APP;TNXB;INSR;CAV1;PECAM1;PIK3R3;ITGA5;TGFBR2;ENG*
Positive Regulation Of Phosphorylation (GO:0042327)	*APP;FLT1;INSR;PECAM1;PIK3R3;ITGA6;ENG*
Positive Regulation Of GTPase Activity (GO:0043547)	*DOCK6;DOCK9;ARHGAP29;ITGA6;RAPGEF5;AGRN;RASGRP3*
Regulation Of Epithelial To Mesenchymal Transition Involved In Endocardial Cushion Formation (GO:1905005)	*ENG;TGFBR2*
Regulation Of Extracellular Matrix Constituent Secretion (GO:0003330)	*NOTCH1;RGCC*
Cardiac Atrium Development (GO:0003230)	*NOTCH1;ENG*
Ventricular Trabecula Myocardium Morphogenesis (GO:0003222)	*NOTCH1;ENG*
Positive Regulation Of Multicellular Organismal Process (GO:0051240)	*RGCC;TNXB;EPAS1;CAV1;ZBTB16;MMRN2;GRB10;LOXL2;TGFBR2*
Artery Morphogenesis (GO:0048844)	*NOTCH1;TGFBR2;ENG*
Positive Regulation Of Hydrolase Activity (GO:0051345)	*DOCK6;DOCK9;ITGA6;RAPGEF5;AGRN;RASGRP3*
Cell-Cell Junction Organization (GO:0045216)	*CDH5;CTNND1;CDH13;LIMS2*
Phagocytosis (GO:0006909)	*GSN;CD93;PECAM1;DOCK1*
Cardiac Muscle Tissue Morphogenesis (GO:0055008)	*NOTCH1;S1PR1;ENG*
Endodermal Cell Differentiation (GO:0035987)	*COL4A2;LAMB1;ITGA5*

Cell-Cell Junction Assembly (GO:0007043)	*CDH5;CTNND1;CDH13;CD9*
Positive Regulation Of Cell-Substrate Adhesion (GO:0010811)	*CDH13;NEDD9;DOCK1;LIMS2*
Positive Regulation Of Extracellular Matrix Assembly (GO:1901203)	*PHLDB1;RGCC*
Epithelial To Mesenchymal Transition Involved In Endocardial Cushion Formation (GO:0003198)	*NOTCH1;ENG*
Hyaluronan Catabolic Process (GO:0030214)	*CEMIP2;HYAL2*
Monocyte Activation (GO:0042117)	*HYAL2;DYSF*
Receptor-Mediated Endocytosis (GO:0006898)	*CALCRL;CAV1;INSR;CD9;HSPG2*
Negative Regulation Of Cell Adhesion (GO:0007162)	*NOTCH1;PLXND1;PODXL;CDH13*
Regulation Of Sprouting Angiogenesis (GO:1903670)	*CEMIP2;ITGA5;KLF2*
Negative Regulation Of Blood Vessel Endothelial Cell Migration (GO:0043537)	*NOTCH1;RGCC;MMRN2*
Endocytosis (GO:0006897)	*APP;CD93;INSR;CAV1;PECAM1;HSPG2*
Regulation Of BMP Signaling Pathway (GO:0030510)	*CDH5;NOTCH1;FSTL1;ENG*
Negative Regulation Of Protein Autophosphorylation (GO:0031953)	*CAV1;ENG*

Positive Regulation Of Vascular Endothelial Growth Factor Receptor Signaling Pathway (GO:0030949)	*GRB10;ITGA5*
Negative Regulation Of Cell-Cell Adhesion Mediated By Cadherin (GO:2000048)	*NOTCH1;RGCC*
Endoderm Formation (GO:0001706)	*COL4A2;LAMB1;ITGA5*
Positive Regulation Of Substrate Adhesion-Dependent Cell Spreading (GO:1900026)	*NEDD9;DOCK1;LIMS2*
Macrophage Activation (GO:0042116)	*APP;CD93;DYSF*
Transmembrane Receptor Protein Serine/Threonine Kinase Signaling Pathway (GO:0007178)	*CDH5;PXN;PTPRK;TGFBR2;ENG*
Substrate Adhesion-Dependent Cell Spreading (GO:0034446)	*LAMA5;PXN;LAMB1*
Regulation Of Protein Phosphorylation (GO:0001932)	*APP;CDH5;INSR;PECAM1;PIK3R3;PLPP3;ENG*
Negative Regulation Of Cell Motility (GO:2000146)	*ADAM15;MMRN2;NEDD9;PTPRK;ENG*
Regulation Of Ossification (GO:0030278)	*NOTCH1;ZBTB16;S1PR1*
Angiogenesis Involved In Wound Healing (GO:0060055)	*MCAM;CD34*
Neuron Projection Maintenance (GO:1990535)	*APP;INSR*
Diol Metabolic Process (GO:0034311)	*PLPP3;PLPP1*

Positive Regulation Of Chondrocyte Differentiation (GO:0032332)	*ZBTB16;LOXL2*
Heart Looping (GO:0001947)	*NOTCH1;TGFBR2;ENG*
Nervous System Development (GO:0007399)	*APP;GSN;PLXND1;ZBTB16;NAV1;RAPGEF5;KALRN;SHANK3;SPTBN1*
Regulation Of Nitric Oxide Biosynthetic Process (GO:0045428)	*CAV1;INSR;KLF2*
Negative Regulation Of Cell-Substrate Adhesion (GO:0010812)	*NOTCH1;ADAM15;ACTN4*
Negative Regulation Of Cell-Cell Adhesion (GO:0022408)	*NOTCH1;RGCC;PODXL*
Sphingoid Metabolic Process (GO:0046519)	*PLPP3;PLPP1*
Lymphocyte Migration (GO:0072676)	*S1PR1;NEDD9;MSN*
Regulation Of Cell Adhesion (GO:0030155)	*TNXB;PLXND1;PODXL;CDH13;LAMB1*
Branching Morphogenesis Of An Epithelial Tube (GO:0048754)	*COL4A1;TGFBR2;ENG*
Cardiac Ventricle Morphogenesis (GO:0003208)	*NOTCH1;TGFBR2;ENG*
Collagen Fibril Organization (GO:0030199)	*COL18A1;SERPINH1;LOXL2*
Determination Of Heart Left/Right Asymmetry (GO:0061371)	*NOTCH1;TGFBR2;ENG*
Embryonic Heart Tube Morphogenesis (GO:0003143)	*NOTCH1;TGFBR2;ENG*

Regulation Of Cold-Induced Thermogenesis (GO:0120161)	*NOTCH1;EPAS1;CAV1;LAMA4;GRB10*
Dephosphorylation (GO:0016311)	*PTPRB;PTPRM;PTPRK;PLPP3;PLPP1*
Positive Regulation Of Integrin-Mediated Signaling Pathway (GO:2001046)	*LAMB1;LIMS2*
Negative Regulation Of Cardiac Muscle Hypertrophy (GO:0010614)	*NOTCH1;LMNA*
Positive Regulation Of Transcription By RNA Polymerase II (GO:0045944)	*APP;NOTCH1;EPAS1;ETS1;KLF2;PTMS;RGCC;NFIB;HYAL2;CDH13;TCF4;ITGA6;AGRN;ENG*
Axon Guidance (GO:0007411)	*NOTCH1;PLXND1;NFIB;PTPRM;KALRN*
Skeletal System Development (GO:0001501)	*COL18A1;LAMA5;ZBTB16;HYAL2;FBN1*
Cellular Response To Ketone (GO:1901655)	*ADAM15;MSN;AQP1*
Atrioventricular Valve Development (GO:0003171)	*NOTCH1;TGFBR2*
Branching Involved In Blood Vessel Morphogenesis (GO:0001569)	*TGFBR2;ENG*
Cardiac Left Ventricle Morphogenesis (GO:0003214)	*NOTCH1;TGFBR2*
Negative Regulation Of Cell Migration Involved In Sprouting Angiogenesis (GO:0090051)	*NOTCH1;MMRN2*
Cellular Response To Growth Factor Stimulus (GO:0071363)	*NOTCH1;FLT1;SHC1;HYAL2;TGFBR2*
Positive Regulation Of Epithelial Cell Migration (GO:0010634)	*SPARC;PLPP3;ETS1;DOCK1*

Positive Regulation Of Protein Modification Process (GO:0031401)	*APP;CDH5;INSR;PECAM1;PIK3R3;ENG*
Positive Regulation Of Long-Term Synaptic Potentiation (GO:1900273)	*APP;SHANK3*
Hyaluronan Metabolic Process (GO:0030212)	*CEMIP2;HYAL2*
Positive Regulation Of Gene Expression (GO:0010628)	*APP;CDH5;NOTCH1;RGCC;GSN;PIK3R3;MSN;ETS1;CD34*
Negative Regulation Of Multicellular Organismal Process (GO:0051241)	*NOTCH1;RGCC;NFIB;LAMA4;SPRY1;LOXL2*
Cellular Response To Insulin Stimulus (GO:0032869)	*SHC1;INSR;GRB10;PIK3R3*
Positive Regulation Of Pathway-Restricted SMAD Protein Phosphorylation (GO:0010862)	*TNXB;TGFBR2;ENG*
Positive Regulation Of Developmental Process (GO:0051094)	*PHLDB1;TNXB;INSR;ZBTB16;MMRN2;SHANK3*
Adenylate Cyclase-Modulating G Protein-Coupled Receptor Signaling Pathway (GO:0007188)	*CALCRL;ADGRF5;ADCY4;ADGRL4;ADGRL2*
Glycosaminoglycan Catabolic Process (GO:0006027)	*CEMIP2;HYAL2*
Supramolecular Fiber Organization (GO:0097435)	*APP;COL18A1;TNXB;GSN;SERPINH1;LOXL2;CNN3*
Receptor Internalization (GO:0031623)	*CALCRL;CAV1;CD9*
Cartilage Development (GO:0051216)	*NFIB;HYAL2;ZBTB16*

Negative Regulation Of MAPK Cascade (GO:0043409)	*CAV1;HYAL2;TIMP3;SPRY1*
Neuron Projection Organization (GO:0106027)	*APP;INSR*
Nuclear Migration (GO:0007097)	*LMNA;SYNE2*
Positive Regulation Of Lymphocyte Migration (GO:2000403)	*APP;NEDD9*
Heart Development (GO:0007507)	*NOTCH1;INSR;ECE1;FBN1;TGFBR2*
Cell-Cell Adhesion Via Plasma-Membrane Adhesion Molecules (GO:0098742)	*CDH5;PECAM1;PTPRM;CDH13;TGFBR2*
Regulation Of Lipid Metabolic Process (GO:0019216)	*CAV1;ADGRF5;PLPP1*
Positive Regulation Of Metabolic Process (GO:0009893)	*EPAS1;CAV1;INSR;GRB10*
Regulation Of Establishment Of Cell Polarity (GO:2000114)	*CDH5;GSN*
T Cell Migration (GO:0072678)	*S1PR1;MSN*
Positive Regulation Of Cartilage Development (GO:0061036)	*ZBTB16;LOXL2*
Positive Regulation Of DNA-templated Transcription (GO:0045893)	*APP;NOTCH1;EPAS1;SHC1;ZBTB16;ETS1;KLF2;PTMS;RGCC;NFIB;HYAL2;CDH13;TCF4;ITGA6;AGRN;ENG*
Regulation Of Transforming Growth Factor Beta Receptor Signaling Pathway (GO:0017015)	*TNXB;CAV1;SPRY1;ENG*

Cardiac Atrium Morphogenesis (GO:0003209)	*NOTCH1;ENG*
Renal Absorption (GO:0070293)	*GSN;HYAL2*
Positive Regulation Of Extracellular Matrix Organization (GO:1903055)	*TNXB;RGCC*
Endothelial Cell Proliferation (GO:0001935)	*CD34;LOXL2*
Mesenchyme Morphogenesis (GO:0072132)	*NOTCH1;ENG*
Myoblast Fusion (GO:0007520)	*CD9;DOCK1*
Regulation Of Cell-Cell Adhesion Mediated By Cadherin (GO:2000047)	*NOTCH1;RGCC*
Regulation Of MAP Kinase Activity (GO:0043405)	*FLT1;INSR;HYAL2;SPRY1*
Heart Morphogenesis (GO:0003007)	*NOTCH1;INSR;ENG*
Regulation Of Integrin-Mediated Signaling Pathway (GO:2001044)	*LAMB1;LIMS2*
Atrioventricular Valve Morphogenesis (GO:0003181)	*NOTCH1;TGFBR2*
Cardiac Ventricle Development (GO:0003231)	*NOTCH1;ENG*
Sphingosine Metabolic Process (GO:0006670)	*PLPP3;PLPP1*
Regulation Of Extrinsic Apoptotic Signaling Pathway (GO:2001236)	*HYAL2;CAV1;ITGA6*
Homophilic Cell Adhesion Via Plasma Membrane Adhesion Molecules (GO:0007156)	*PECAM1;PTPRM;CDH13*

		Regulation Of Pathway-Restricted SMAD Protein Phosphorylation (GO:0060393)	*TNXB;TGFBR2;ENG*
		Cellular Response To Tumor Necrosis Factor (GO:0071356)	*PLVAP;HYAL2;ACTN4;YBX3*
		Endothelial Cell Development (GO:0001885)	*PECAM1;MSN*
		Myoblast Differentiation (GO:0045445)	*NOTCH1;EPAS1*
COL4A1^{+} fibroblasts			
Protein digestion and absorption	*COL18A1;COL16A1;COL1A1;COL3A1;COL1A2;COL4A2;COL5A1;COL4A1;COL6A2;COL5A3;COL6A1;COL5A2;COL6A3;SLC38A2*	Extracellular Matrix Organization (GO:0030198)	*COL18A1;COL16A1;ADAMTS12;NID1;NID2;LOXL2;COL1A1;ADAMTS14;COL3A1;COL1A2;COL4A2;COL5A1;COL4A1;COL5A3;PXDN;COL5A2*
ECM-receptor interaction	*COL1A1;COL1A2;COL4A2;COL4A1;LAMB2;COL6A2;LAMA4;ITGA1;COL6A1;COL6A3;LAMB1;AGRN*	Extracellular Structure Organization (GO:0043062)	*COL1A1;ADAMTS14;COL3A1;COL16A1;COL1A2;COL4A2;COL5A1;COL4A1;COL5A3;PXDN;COL5A2;ADAMTS12*
Focal adhesion	*PDGFRB;LAMB2;LAMA4;ITGA1;LAMB1;COL1A1;COL1A2;COL4A2;COL4A1;COL6A2;COL6A1;COL6A3;PPP1R12B;MYL9*	External Encapsulating Structure Organization (GO:0045229)	*COL1A1;ADAMTS14;COL3A1;COL16A1;COL1A2;COL4A2;COL5A1;COL4A1;COL5A3;PXDN;COL5A2;ADAMTS12*
Human papillomavirus infection	*PDGFRB;NOTCH3;JAG1;LAMB2;LAMA4;ITGA1;LAMB1;COL1A1;HEYL;COL1A2;COL4A2;COL4A1;COL6A2;COL6A1;COL6A3*	Collagen Fibril Organization (GO:0030199)	*COL1A1;COL18A1;COL3A1;COL1A2;COL5A1;COL5A3;COL5A2;PXDN;LOXL2*
PI3K-Akt signaling pathway	*PDGFRB;LAMB2;LAMA4;ITGA1;LAMB1;OSMR;COL1A1;COL1A2;COL4A2;COL4A1;COL6A2;COL6A1;COL6A3*	Supramolecular Fiber Organization (GO:0097435)	*COL18A1;TPM2;TPM1;LOXL2;COL1A1;ADAMTS14;MYO1B;COL3A1;COL1A2;COL5A1;COL5A3;PXDN;COL5A2;MAP4;MYL9*

Amoebiasis	*COL1A1;COL3A1;COL1A2;COL4A2;COL4A1;LAMB2;LAMA4;LAMB1*	Skin Development (GO:0043588)	*COL1A1;COL3A1;JAG1;COL1A2;COL5A1;COL5A3;COL5A2*
Relaxin signaling pathway	*ACTA2;COL1A1;COL3A1;COL1A2;COL4A2;COL4A1*	Basement Membrane Organization (GO:0071711)	*COL4A1;PXDN;NID1;NID2*
Small cell lung cancer	*COL4A2;COL4A1;LAMB2;LAMA4;LAMB1*	Regulation Of Basement Membrane Organization (GO:0110011)	*LAMB2;LAMB1;NID1*
AGE-RAGE signaling pathway in diabetic complications	*COL1A1;COL3A1;COL1A2;COL4A2;COL4A1*	Muscle Contraction (GO:0006936)	*GJC1;SMTN;TPM2;TPM1;UTRN;CACNA1H*
Vascular smooth muscle contraction	*ACTA2;CALD1;PPP1R12B;MYL9;PRKG1*	Muscle Organ Development (GO:0007517)	*TAGLN;SMTN;COL6A3;UTRN;CACNA1H*
Pathways in cancer	*PDGFRB;NOTCH3;HEYL;JAG1;COL4A2;COL4A1;LAMB2;LAMA4;LAMB1*	Cell-Matrix Adhesion (GO:0007160)	*COL3A1;ITGA1;THY1;ARHGEF7;ADAMTS12;NID2*
Platelet activation	*COL1A1;COL3A1;COL1A2;PRKG1*	Regulation Of Muscle System Process (GO:0090257)	*TPM1;PPP1R12B;MYL9*
Notch signaling pathway	*NOTCH3;HEYL;JAG1*	Positive Regulation Of Integrin-Mediated Signaling Pathway (GO:2001046)	*LAMB2;LAMB1;NID1*
Regulation of actin cytoskeleton	*PDGFRB;ITGA1;PPP1R12B;ARHGEF7;MYL9*	Positive Regulation Of Muscle Cell Differentiation (GO:0051149)	*LAMB2;LAMB1;NID1*
Hypertrophic cardiomyopathy	*TPM2;ITGA1;TPM1*	Eye Morphogenesis (GO:0048592)	*COL5A1;COL5A2;FBN1*
Dilated cardiomyopathy	*TPM2;TPM1;ITGA1*	Regulation Of Integrin-Mediated Signaling Pathway (GO:2001044)	*LAMB2;LAMB1;NID1*
		Eye Development (GO:0001654)	*COL5A1;COL5A2;HIPK2;FBN1*
		Regulation Of Extracellular Matrix Organization (GO:1903053)	*LAMB2;LAMB1;NID1*

Regulation Of Muscle Cell Differentiation (GO:0051147)	*LAMB2;LAMB1;NID1*
Regulation Of Muscle Contraction (GO:0006937)	*TPM1;PPP1R12B;MYL9*
Endodermal Cell Differentiation (GO:0035987)	*COL4A2;COL6A1;LAMB1*
Endoderm Formation (GO:0001706)	*COL4A2;COL6A1;LAMB1*
Skin Morphogenesis (GO:0043589)	*COL1A1;COL1A2*
Integrin-Mediated Signaling Pathway (GO:0007229)	*COL3A1;COL16A1;ITGA1;THY1*
Positive Regulation Of Cell Adhesion (GO:0045785)	*LAMB2;TPM1;LAMB1;NID1*
Odontogenesis (GO:0042476)	*COL1A1;COL1A2;LAMB1*
Notch Signaling Pathway (GO:0007219)	*NOTCH3;HEYL;JAG1*
Notch Signaling Involved In Heart Development (GO:0061314)	*HEYL;JAG1*
Cardiac Ventricle Morphogenesis (GO:0003208)	*HEYL;JAG1;TPM1*
Positive Regulation Of Cell-Matrix Adhesion (GO:0001954)	*COL16A1;THY1;UTRN*
Myofibril Assembly (GO:0030239)	*PDGFRB;TPM1;MYL9*
Positive Regulation Of Epithelial To Mesenchymal Transition (GO:0010718)	*COL1A1;JAG1;LOXL2*
Regulation Of Vascular Associated Smooth Muscle Cell Migration (GO:1904752)	*TPM1;PRKG1*

Positive Regulation Of Cell Differentiation (GO:0045597)	*COL1A1;HEYL;LAMB2;LAMB1;NID1;LOXL2*
Negative Regulation Of Smooth Muscle Cell Migration (GO:0014912)	*TPM1;PRKG1*
Aorta Development (GO:0035904)	*PDGFRB;JAG1*
Cell Surface Receptor Signaling Pathway Involved In Heart Development (GO:0061311)	*HEYL;JAG1*
Negative Regulation Of Vascular Associated Smooth Muscle Cell Proliferation (GO:1904706)	*TPM1;PRKG1*
Aorta Morphogenesis (GO:0035909)	*PDGFRB;JAG1*
Positive Regulation Of Phosphoprotein Phosphatase Activity (GO:0032516)	*PDGFRB;ITGA1*
Pulmonary Valve Morphogenesis (GO:0003184)	*HEYL;JAG1*
Positive Regulation Of Focal Adhesion Assembly (GO:0051894)	*COL16A1;THY1*
Plasma Membrane Bounded Cell Projection Organization (GO:0120036)	*TPM1;MICALL2;LAMB1;MAP4*
Circulatory System Development (GO:0072359)	*HEYL;COL3A1;PDLIM7;FBN1*
Regulation Of Cell-Matrix Adhesion (GO:0001952)	*JAG1;THY1;UTRN*
Regulation Of Cell Migration (GO:0030334)	*PDGFRB;COL1A1;JAG1;DAAM2;TPM1;LAMB1;THY1*

COL11A1^{+} fibroblasts			
ECM-receptor interaction	*LAMB2;TNC;FN1;LAMB1;THBS2;THBS1;THBS4;THBS3;COMP;COL1A1;COL1A2;COL6A2;COL6A1;COL6A3;ITGAV*	Extracellular Matrix Organization (GO:0030198)	*POSTN;COL15A1;COL16A1;ECM2;COL14A1;LUM;ELN;MMP2;COL11A1;COL12A1;DPT;LOXL1;COL1A1;SMOC2;ADAMTS2;MMP14;COL3A1;CCDC80;COL1A2;COL5A1;CTSK;COL5A2;SERPINH1;COL8A1*
Protein digestion and absorption	*COL15A1;COL16A1;COL14A1;ELN;COL11A1;COL12A1;COL1A1;COL3A1;COL1A2;COL5A1;COL6A2;COL5A2;COL6A1;COL8A1;COL6A3*	Collagen Fibril Organization (GO:0030199)	*COL14A1;LUM;COL11A1;COL12A1;DPT;LOXL1;COL1A1;ADAMTS2;COL3A1;COL1A2;COL5A1;COL5A2;SERPINH1*
Focal adhesion	*PDGFRB;LAMB2;TNC;FN1;LAMB1;THBS2;THBS1;THBS4;THBS3;COMP;COL1A1;COL1A2;COL6A2;COL6A1;COL6A3;ITGAV;MYL9;BCAR1*	Extracellular Structure Organization (GO:0043062)	*POSTN;COL15A1;COL16A1;ECM2;COL14A1;MMP2;COL11A1;COL1A1;SMOC2;ADAMTS2;MMP14;COL3A1;CCDC80;COL1A2;COL5A1;COL5A2;COL8A1*
Human papillomavirus infection	*PDGFRB;LAMB2;TNC;FN1;LAMB1;THBS2;THBS1;FOXO1;THBS4;THBS3;COMP;COL1A1;COL1A2;DLG2;COL6A2;COL6A1;COL6A3;ITGAV*	External Encapsulating Structure Organization (GO:0045229)	*POSTN;COL15A1;COL16A1;ECM2;COL14A1;MMP2;COL11A1;COL1A1;SMOC2;ADAMTS2;MMP14;COL3A1;CCDC80;COL1A2;COL5A1;COL5A2;COL8A1*
PI3K-Akt signaling pathway	*PDGFRB;LAMB2;TNC;FN1;LAMB1;OSMR;THBS2;THBS1;THBS4;THBS3;COMP;COL1A1;COL1A2;COL6A2;COL6A1;COL6A3;ITGAV;FGFR1*	Endodermal Cell Differentiation (GO:0035987)	*MMP14;COL11A1;MMP2;COL12A1;COL6A1;FN1;COL8A1;ITGAV;LAMB1;INHBA*
AGE-RAGE signaling pathway in diabetic complications	*COL1A1;EGR1;COL3A1;COL1A2;MMP2;SERPINE1;FN1;F3;FOXO1*	Supramolecular Fiber Organization (GO:0097435)	*COL14A1;LUM;TPM2;COL11A1;COL12A1;TPM1;DPT;LTBP2;LOXL1;CNN3;COL1A1;ADAMTS2;COL3A1;COL1A2;COL5A1;COL5A2;SERPINH1;FAT1;EMILIN1;MYL9;BCAR1*

Proteoglycans in cancer	*COL1A1;COL1A2;PLAU;LUM;MMP2;FN1;TIMP3;ITGAV;THBS1;DCN;FGFR1*	Endoderm Formation (GO:0001706)	*MMP14;MMP2;COL12A1;COL6A1;FN1;COL8A1;ITGAV;LAMB1;INHBA*
Malaria	*COMP;LRP1;THBS2;THBS1;THBS4;THBS3*	Regulation Of Cell Migration (GO:0030334)	*PDGFRB;LRRC15;CEMIP;SERPINE1;TPM1;TNC;LAMB1;THY1;PODN;SULF1;THBS1;COL1A1;MMP14;SFRP2;CXCL12;PLAU;DPYSL3;CDH11;EMILIN1;BCAR1*
Phagosome	*COMP;MRC2;C1R;ITGAV;THBS2;THBS1;THBS4;THBS3*	Negative Regulation Of Cell Migration (GO:0030336)	*LRP1;SERPINE1;TPM1;THY1;PODN;SULF1;PTPRG;GJA1;COL3A1;SFRP2;DPYSL3;CDH11;EMILIN1*
Complement and coagulation cascades	*C1S;CFH;PLAU;C1R;SERPINE1;F3*	Skeletal System Development (GO:0001501)	*COMP;KIAA1217;COL1A1;GJA1;MMP14;COL1A2;CDH11;SULF1;FBN1;FGFR1;SULF2*
Amoebiasis	*COL1A1;COL3A1;COL1A2;LAMB2;FN1;LAMB1*	Negative Regulation Of Angiogenesis (GO:0016525)	*SPARC;SERPINF1;SERPINE1;EMILIN1;THBS2;SULF1;THBS1;DCN;THBS4*
Regulation of actin cytoskeleton	*PDGFRB;ENAH;CXCL12;FN1;ITGAV;MYL9;BCAR1;FGFR1*	Negative Regulation Of Cell Motility (GO:2000146)	*SFRP2;SERPINE1;DPYSL3;TPM1;CDH11;FBLN1;EMILIN1;THY1;PODN;SULF1*
TGF-beta signaling pathway	*INHBA;NBL1;THBS1;DCN;FBN1*	Negative Regulation Of Blood Vessel Morphogenesis (GO:2000181)	*SPARC;SERPINF1;EMILIN1;THBS2;SULF1;THBS1;DCN;THBS4*
Leukocyte transendothelial migration	*CXCL12;MMP2;THY1;MYL9;BCAR1*	Cell-Matrix Adhesion (GO:0007160)	*COL3A1;ECM2;CDH11;FN1;ITGBL1;EMILIN1;ITGAV;THY1;THBS3*
Bacterial invasion of epithelial cells	*FN1;SEPTIN11;DNM1;BCAR1*	Integrin-Mediated Signaling Pathway (GO:0007229)	*COL3A1;COL16A1;FN1;ITGBL1;ITGAV;FBLN1;THY1;BCAR1*
Small cell lung cancer	*LAMB2;FN1;ITGAV;LAMB1*	Positive Regulation Of Cell Adhesion (GO:0045785)	*ECM2;CCDC80;SFRP2;CXCL12;LAMB2;TPM1;ITGAV;LAMB1*
Prostate cancer	*PDGFRB;PLAU;FOXO1;FGFR1*	Regulation Of Angiogenesis (GO:0045765)	*SMOC2;SPARC;SFRP2;SERPINF1;SERPINE1;EMILIN1;THBS2;SULF1;THBS1;DCN;THBS4*

Pathways in cancer	*PDGFRB;CXCL12;LAMB2;MMP2;FN1;ITGAV;LAMB1;FOXO1;GLI3;FGFR1*	Negative Regulation Of Smooth Muscle Cell Migration (GO:0014912)	*LRP1;SERPINE1;TPM1;PRKG1*
		Negative Regulation Of Signal Transduction (GO:0009968)	*SFRP4;MMP14;SFRP2;LRP1;ENPP1;EMILIN1;SULF1;THBS1;GLI3;DCN;SULF2*
		Negative Regulation Of Peptidase Activity (GO:0010466)	*LRP1;SERPINF1;SERPINE1;TIMP2;SERPINH1;TIMP3*
		Positive Regulation Of Cell Migration (GO:0030335)	*PDGFRB;COL1A1;LRRC15;CEMIP;MMP14;CXCL12;PLAU;FN1;LAMB1;THBS1;BCAR1*
		Positive Regulation Of Cell Motility (GO:2000147)	*PDGFRB;COL1A1;LRRC15;CEMIP;MMP14;CXCL12;PLAU;LAMB1;THBS1;BCAR1*
		Negative Regulation Of Plasminogen Activation (GO:0010757)	*PLAU;SERPINE1;THBS1*
		Regulation Of Fibroblast Growth Factor Receptor Signaling Pathway (GO:0040036)	*SMOC2;SULF1;THBS1;SULF2*
		Regulation Of Smooth Muscle Cell Migration (GO:0014910)	*PDGFRB;LRP1;PLAU;SERPINE1*
		Skin Development (GO:0043588)	*COL1A1;COL3A1;COL1A2;COL5A1;COL5A2;FGFR1*
		Regulation Of Cell Adhesion (GO:0030155)	*CXCL12;PLAU;LAMB2;TPM1;TNC;FBLN1;ITGAV;LAMB1*
		Positive Regulation Of Blood Coagulation (GO:0030194)	*PLAU;SERPINE1;EMILIN1;THBS1*
		Peptide Cross-Linking (GO:0018149)	*COL3A1;FN1;THBS1;FGFR1*

Regulation Of Blood Coagulation (GO:0030193)	*PLAU;SERPINE1;EMILIN1;THBS1*
Negative Regulation Of Fibrinolysis (GO:0051918)	*PLAU;SERPINE1;THBS1*
Positive Regulation Of Coagulation (GO:0050820)	*SERPINE1;EMILIN1;THBS1*
Positive Regulation Of Hemostasis (GO:1900048)	*SERPINE1;EMILIN1;THBS1*
Chordate Embryonic Development (GO:0043009)	*KIAA1217;COL1A1;SULF1;SULF2; FGFR1*
Actin Filament Organization (GO:0007015)	*ENAH;TPM2;DPYSL3;TPM1;FAT1; BCAR1;CNN3*
Negative Regulation Of Fibroblast Growth Factor Receptor Signaling Pathway (GO:0040037)	*SULF1;THBS1;SULF2*
Embryonic Skeletal System Development (GO:0048706)	*COL1A1;KIAA1217;SULF1;SULF2*
Negative Regulation Of Endopeptidase Activity (GO:0010951)	*SERPINF1;SERPINE1;TIMP2;SERPINH1;TIMP3*
Regulation Of Vascular Associated Smooth Muscle Cell Proliferation (GO:1904705)	*GJA1;MMP2;TPM1;PRKG1*
Regulation Of Endopeptidase Activity (GO:0052548)	*SERPINF1;SERPINE1;TIMP2;SERPINH1;TIMP3*
Negative Regulation Of Protein Processing (GO:0010955)	*PLAU;SERPINE1;THBS1*
Proteolysis (GO:0006508)	*ADAMTS2;MMP14;CFH;PLAU;CTSK;MMP2;ADAM12;HTRA1;PCOLCE;AEBP1*

Negative Regulation Of Cellular Response To Growth Factor Stimulus (GO:0090288)	*EMILIN1;SULF1;NBL1;THBS1;SULF2*
Odontogenesis (GO:0042476)	*COL1A1;COL1A2;LAMB1;INHBA*
Regulation Of Fibrinolysis (GO:0051917)	*PLAU;SERPINE1;THBS1*
Regulation Of Plasminogen Activation (GO:0010755)	*PLAU;SERPINE1;THBS1*
Positive Regulation Of Wound Healing (GO:0090303)	*SMOC2;SERPINE1;EMILIN1;THBS1*
Bone Development (GO:0060348)	*SFRP4;GJA1;SULF1;SULF2*
Regulation Of Cellular Response To Growth Factor Stimulus (GO:0090287)	*SFRP4;SULF1;FSTL1*
Eye Morphogenesis (GO:0048592)	*COL5A1;COL5A2;FBN1*
Positive Regulation Of Smooth Muscle Cell Proliferation (GO:0048661)	*PDGFRB;GJA1;MMP2;THBS1*
Positive Regulation Of Endothelial Cell Migration (GO:0010595)	*SMOC2;SPARC;THBS1;BCAR1;FGFR1*
Positive Regulation Of Cellular Process (GO:0048522)	*PDGFRB;LAMB2;TPM1;FN1;LAMB1;OSMR;THBS1;MMP14;SFRP2;CXCL12;ITGAV;CCN2;FGFR1*
Regulation Of Endothelial Cell Migration (GO:0010594)	*SMOC2;SPARC;THBS1;DCN;BCAR1*
Chondrocyte Development (GO:0002063)	*SULF1;SULF2*
Positive Regulation Of Extrinsic Apoptotic Signaling Pathway	*TIMP3;THBS1*

Via Death Domain Receptors (GO:1902043)	
Regulation Of Basement Membrane Organization (GO:0110011)	*LAMB2;LAMB1*
Regulation Of Extracellular Matrix Organization (GO:1903053)	*LRP1;LAMB2;LAMB1*
Positive Regulation Of Vascular Endothelial Growth Factor Production (GO:0010575)	*RORA;SULF1;SULF2*
Negative Regulation Of Membrane Protein Ectodomain Proteolysis (GO:0051045)	*TIMP2;TIMP3*
Regulation Of Metallopeptidase Activity (GO:1905048)	*LRP1;TIMP2*
Maintenance Of Protein Location In Extracellular Region (GO:0071694)	*NBL1;FBN1*
Regulation Of Monocyte Chemotaxis (GO:0090025)	*CXCL12;SERPINE1;NBL1*
Negative Regulation Of Protein Phosphorylation (GO:0001933)	*SFRP2;LRP1;ENPP1;FBLN1;EMILIN1;THY1*
Negative Regulation Of Cell Differentiation (GO:0045596)	*SFRP2;COL5A1;COL5A2;ENPP1;RORA;ITGAV;FOXO1*
Regulation Of Vascular Endothelial Growth Factor Production (GO:0010574)	*RORA;SULF1;SULF2*
Positive Regulation Of Response To External Stimulus (GO:0032103)	*PDGFRB;PLAU;SERPINE1;NFKBIZ;EMILIN1;THBS1*

Positive Regulation Of Vasculature Development (GO:1904018)	*SMOC2;SFRP2;SERPINE1;EMILIN1;THBS1*
Regulation Of Vascular Wound Healing (GO:0061043)	*SMOC2;SERPINE1*
Positive Regulation Of Calcium Ion Import (GO:0090280)	*PDGFRB;CXCL12*
Positive Regulation Of Gluconeogenesis (GO:0045722)	*NNMT;FOXO1*
Apoptotic Cell Clearance (GO:0043277)	*LRP1;ITGAV;THBS1*
Cell-Substrate Junction Assembly (GO:0007044)	*CDH11;FN1;THY1*
Positive Regulation Of Cell Differentiation (GO:0045597)	*COL1A1;GJA1;SFRP2;LAMB2;LAMB1;CCN2;NBL1;FGFR1*
Transmembrane Receptor Protein Tyrosine Kinase Signaling Pathway (GO:0007169)	*PDGFRB;MMP2;SULF1;FOXO1;BCAR1;FGFR1;PTPRG;SULF2*
Positive Regulation Of Wnt Signaling Pathway (GO:0030177)	*COL1A1;SFRP4;SFRP2;SULF1;SULF2*
Regulation Of Wnt Signaling Pathway (GO:0030111)	*SFRP4;SFRP2;LRP1;SULF1;SULF2*
Sensory Organ Morphogenesis (GO:0090596)	*COL5A2;GLI3*
Skin Morphogenesis (GO:0043589)	*COL1A1;COL1A2*
Positive Regulation Of Extracellular Matrix Assembly (GO:1901203)	*PHLDB1;EMILIN1*
Integrin Activation (GO:0033622)	*CXCL12;FN1*

Regulation Of Cell Growth (GO:0001558)	*GJA1;MMP14;SFRP2;TNC;ENPP1;INHBA;BCAR1*
Negative Regulation Of Fat Cell Differentiation (GO:0045599)	*ENPP1;RORA;FOXO1*
Positive Regulation Of Phosphatidylinositol 3-Kinase Signaling (GO:0014068)	*PDGFRB;FN1;DCN;FGFR1*
Negative Regulation Of Epithelial Cell Proliferation (GO:0050680)	*SPARC;SFRP2;SULF1;THBS1*
Negative Regulation Of Epithelial Cell Migration (GO:0010633)	*THBS1;DCN;PTPRG*
Actin Filament Bundle Assembly (GO:0051017)	*LIMA1;CALD1;DPYSL3*
Actin Filament Bundle Organization (GO:0061572)	*LIMA1;CALD1;DPYSL3*
Regulation Of Cell Adhesion Mediated By Integrin (GO:0033628)	*SFRP2;PLAU;SERPINE1*
Sequestering Of Extracellular Ligand From Receptor (GO:0035581)	*NBL1;FBN1*
Positive Regulation Of Cellular Biosynthetic Process (GO:0031328)	*PDGFRB;EGR1;SMOC2;NNMT;THBS1;FOXO1*
Positive Regulation Of Angiogenesis (GO:0045766)	*SMOC2;SFRP2;SERPINE1;EMILIN1;THBS1*
Negative Regulation Of Cellular Process (GO:0048523)	*SFRP4;GJA1;SFRP2;CCN5;ENPP1;FBLN1;EMILIN1;CCN2;INHBA;PODN;THBS1*

Enzyme-Linked Receptor Protein Signaling Pathway (GO:0007167)	*PDGFRB;LRP1;BCAR1;FGFR1;PTPRG*
Regulation Of Fat Cell Differentiation (GO:0045598)	*SFRP2;ENPP1;RORA;FOXO1*
Regulation Of BMP Signaling Pathway (GO:0030510)	*SFRP4;SULF1;NBL1;FSTL1*
Negative Regulation Of Pathway-Restricted SMAD Protein Phosphorylation (GO:0060394)	*LRP1;EMILIN1*
Regulation Of Lipid Transport (GO:0032368)	*LRP1;ITGAV*
Regulation Of Phospholipase Activity (GO:0010517)	*LRP1;FGFR1*
Substrate Adhesion-Dependent Cell Spreading (GO:0034446)	*FN1;ITGAV;LAMB1*
Response To Cytokine (GO:0034097)	*COL3A1;DPYSL3;TIMP2;TIMP3;OSMR*
Negative Regulation Of Endothelial Cell Proliferation (GO:0001937)	*SPARC;SULF1;THBS1*
Aortic Valve Morphogenesis (GO:0003180)	*ELN;CDH11;EMILIN1*
Regulation Of Muscle System Process (GO:0090257)	*TPM1;MYL9*
Regulation Of Phospholipase C Activity (GO:1900274)	*PDGFRB;FGFR1*
Lipoprotein Transport (GO:0042953)	*VMP1;LRP1*
Circulatory System Development (GO:0072359)	*GJA1;COL3A1;FN1;PDLIM7;FBN1*

Phosphate Ion Homeostasis (GO:0055062)	*SFRP4;ENPP1*
Regulation Of Vascular Associated Smooth Muscle Cell Migration (GO:1904752)	*TPM1;PRKG1*
Positive Regulation Of Integrin-Mediated Signaling Pathway (GO:2001046)	*LAMB2;LAMB1*
Fibrinolysis (GO:0042730)	*PLAU;SERPINE1*
Lipoprotein Localization (GO:0044872)	*VMP1;LRP1*
Positive Regulation Of Signal Transduction (GO:0009967)	*SMOC2;LAMB2;CTSK;ITGAV;LAMB1;SULF1;SULF2*
Response To Amyloid-Beta (GO:1904645)	*GJA1;LRP1;MMP2*
Positive Regulation Of Receptor-Mediated Endocytosis (GO:0048260)	*SFRP4;SERPINE1;F3*
Glomerular Filtration (GO:0003094)	*SULF1;SULF2*
Heparan Sulfate Proteoglycan Metabolic Process (GO:0030201)	*SULF1;SULF2*
Positive Regulation Of Chemotaxis (GO:0050921)	*PDGFRB;SMOC2;THBS1*
Extracellular Matrix Disassembly (GO:0022617)	*MMP14;CTSK;MMP2*
Regulation Of Cell-Substrate Adhesion (GO:0010810)	*ECM2;CCDC80;THY1*
Zymogen Activation (GO:0031638)	*MMP14;C1R;PLAU*

Regulation Of Phosphatidylinositol 3-Kinase Signaling (GO:0014066)	*PDGFRB;FN1;DCN;FGFR1*
Regulation Of Endothelial Cell Chemotaxis (GO:2001026)	*SMOC2;THBS1*
Aorta Development (GO:0035904)	*PDGFRB;LRP1*
Positive Regulation Of Endothelial Cell Chemotaxis (GO:2001028)	*SMOC2;FGFR1*
Cellular Component Disassembly (GO:0022411)	*MMP14;CTSK;MMP2*
Myofibril Assembly (GO:0030239)	*PDGFRB;TPM1;MYL9*
Regulation Of Endothelial Cell Proliferation (GO:0001936)	*SPARC;SULF1;THBS1;THBS4*
Regulation Of Intrinsic Apoptotic Signaling Pathway In Response To DNA Damage (GO:1902229)	*CXCL12;SFRP2*
Platelet-Derived Growth Factor Receptor Signaling Pathway (GO:0048008)	*PDGFRB;BCAR1*
Renal Filtration (GO:0097205)	*SULF1;SULF2*
Positive Regulation Of Glucose Metabolic Process (GO:0010907)	*NNMT;FOXO1*
Positive Regulation Of Muscle Cell Differentiation (GO:0051149)	*LAMB2;LAMB1*
Negative Regulation Of ERK1 And ERK2 Cascade (GO:0070373)	*TIMP3;FBLN1;EMILIN1*

		Positive Regulation Of Epithelial Cell Migration (GO:0010634)	*SMOC2;SPARC;THBS1;BCAR1*
		Cell Chemotaxis (GO:0060326)	*PDGFRB;CXCL12;BCAR1*
		Regulation Of Stem Cell Proliferation (GO:0072091)	*GJA1;FBLN1*
		Regulation Of Transforming Growth Factor Beta Production (GO:0071634)	*FN1;ITGAV*
		Ruffle Organization (GO:0031529)	*LIMA1;TPM1*
		Positive Regulation Of Stem Cell Proliferation (GO:2000648)	*GJA1;LTBP2*
		Regulation Of Apoptotic Process (GO:0042981)	*COMP;SFRP4;EGR1;SFRP2;ANXA5;HTRA1;EMILIN1;CCNL2;FSTL1;THBS1;FOXO1;BCAR1*
Lymph endothelial cells			
Rap1 signaling pathway	*VAV3;AFDN;FLT4;PLCG1;IGF1;EFNA5;RAPGEF5;BCAR1;EPHA2;RASGRP3*	Regulation Of Angiogenesis (GO:0045765)	*CDH5;CLDN5;JUP;RHOJ;ECSCR;HSPB1;PTPRM;EMILIN1;PLCG1;HSPG2;EPHA2*
Ras signaling pathway	*AFDN;SHC1;FLT4;PLCG1;IGF1;EFNA5;RAPGEF5;GNG11;EPHA2;RASGRP3*	Positive Regulation Of Cell Motility (GO:2000147)	*TJP1;CDH5;CCL21;CAVIN1;FLT4;PDPN;ADAM10;IGF1;PPM1F;BCAR1;EPHA2*
Focal adhesion	*VAV3;SHC1;FLT4;LAMA4;IGF1;LAMC1;ITGA5;BCAR1;ITGA9*	Positive Regulation Of Cell Migration (GO:0030335)	*TJP1;CDH5;DOCK5;FLT4;PDPN;ADAM10;PLCG1;IGF1;PPM1F;BCAR1;EPHA2*
PI3K-Akt signaling pathway	*FLT4;LAMA4;PPP2R5A;IGF1;LAMC1;ITGA5;EFNA5;GNG11;EPHA2;HSP90B1;ITGA9*	Lymphatic Endothelial Cell Differentiation (GO:0060836)	*TIE1;PDPN;PROX1*
Leukocyte transendothelial migration	*VAV3;CDH5;CLDN5;AFDN;PLCG1;BCAR1*	Actomyosin Structure Organization (GO:0031032)	*TJP1;EPB41L2;TMOD2;MYO18A;PGM5;MYH10*
ECM-receptor interaction	*LAMA4;LAMC1;ITGA5;HSPG2;ITGA9*	Positive Regulation Of Angiogenesis (GO:0045766)	*TJP1;CDH5;JUP;HSPB1;EMILIN1;PLCG1;ITGA5*

Epithelial cell signaling in Helicobacter pylori infection	*TJP1;ADAM10;NOD1;PLCG1*	Regulation Of Platelet Aggregation (GO:0090330)	*MMRN1;PDPN;EMILIN1;PRKG1*
		Basement Membrane Organization (GO:0071711)	*FLRT2;PXDN;NID1*
		Regulation Of Endothelial Cell Migration (GO:0010594)	*FLT4;RHOJ;PTPRM;PROX1;BCAR1;EPHA2*
		Transmembrane Receptor Protein Tyrosine Kinase Signaling Pathway (GO:0007169)	*FLRT2;SHC1;FLT4;TIE1;PILRB;IGF1;EFNA5;KALRN;BCAR1;EPHA2*
		Maintenance Of Blood-Brain Barrier (GO:0035633)	*TJP1;CDH5;CLDN5;LAMC1*
		Positive Regulation Of Platelet Aggregation (GO:1901731)	*MMRN1;PDPN;EMILIN1*
		Negative Regulation Of Apoptotic Process (GO:0043066)	*TBX1;UNC5B;SHC1;FLT4;HSPB1;IGF1;CLU;HSP90B1;TJP1;PDPN;MYO18A;ITGA5;TXNDC5*
		Positive Regulation Of Epithelial Cell Migration (GO:0010634)	*DOCK5;FLT4;PROX1;PLCG1;PPM1F;BCAR1*
		Microtubule Anchoring At Microtubule Organizing Center (GO:0072393)	*DCTN1;BICD1;NINL*
		Positive Regulation Of Supramolecular Fiber Organization (GO:1902905)	*APP;NCKAP1;CCL21;DCTN1;PROX1;CLU*
		Regulation Of ERK1 And ERK2 Cascade (GO:0070372)	*APP;CCL21;SHC1;FLT4;CNKSR3;EMILIN1;IGF1;DUSP6;EPHA2*
		Ras Protein Signal Transduction (GO:0007265)	*NCKAP1;PDPN;ARHGAP29;IGF1;CELSR1;RAPGEF5;RASGRP3*
		Regulation Of Cell Migration (GO:0030334)	*TJP1;CDH5;CLDN5;ROBO4;FLT4;PDPN;ADAM10;EMILIN1;IGF1;PPM1F;BCAR1;EPHA2*

Regulation Of Bicellular Tight Junction Assembly (GO:2000810)	*TJP1;CLDN5;EPHA2*
Cellular Response To Growth Factor Stimulus (GO:0071363)	*TBX1;SHC1;FLT4;HYAL2;HSPB1;PLCG1;BCAR1*
Positive Regulation Of Cell-Substrate Adhesion (GO:0010811)	*DOCK5;CCL21;JUP;TRIOBP;PPM1F*
Regulation Of Receptor-Mediated Endocytosis (GO:0048259)	*CCL21;SGIP1;BICD1;CLU*
Positive Regulation Of Homotypic Cell-Cell Adhesion (GO:0034112)	*MMRN1;PDPN;EMILIN1*
Regulation Of GTPase Activity (GO:0043087)	*DOCK6;VAV3;CCL21;DOCK9;EFNA5;RAPGEF5;PRKG1;RASGRP3*
Regulation Of Cytoskeleton Organization (GO:0051493)	*TJP1;ARHGAP17;CELSR1;EFNA5;PHLDB2;BICD1*
Regulation Of Cell Population Proliferation (GO:0042127)	*KANK2;TBX1;APP;JUP;SHC1;FLT4;ADAM10;IGF1;PROX1;CLU;TJP1;CLDN5;PDPN;IGFBP7;EMILIN1;CD200*
Regulation Of Microtubule-Based Process (GO:0032886)	*CCNL2;EFNA5;BICD1;PHLDB2*
Negative Regulation Of Blood Vessel Morphogenesis (GO:2000181)	*CLDN5;ECSCR;PTPRM;EMILIN1;HSPG2*
Positive Regulation Of Receptor-Mediated Endocytosis (GO:0048260)	*CCL21;SGIP1;BICD1;CLU*
Positive Regulation Of Epithelial Cell Proliferation (GO:0050679)	*TBX1;EGFL7;FLT4;IGF1;LAMC1;PROX1*

Negative Regulation Of Peptidyl-Serine Phosphorylation (GO:0033137)	*PDE4D;CNKSR3;PPM1F*
Regulation Of Anatomical Structure Morphogenesis (GO:0022603)	*TBX1;HECW2;FLT4;EFNA5;PHLDB2;EPHA2*
Positive Regulation Of Endothelial Cell Migration (GO:0010595)	*FLT4;HSPB1;PROX1;PLCG1;BCAR1*
Negative Regulation Of Angiogenesis (GO:0016525)	*CLDN5;ECSCR;PTPRM;EMILIN1;HSPG2*
Regulation Of Epithelial Cell Migration (GO:0010632)	*DOCK5;RHOJ;PLCG1;PPM1F*
Axo-Dendritic Transport (GO:0008088)	*APP;DCTN1*
Positive Regulation Of Antigen Processing And Presentation (GO:0002579)	*CCL21;NOD1*
Positive Regulation Of Dendritic Cell Antigen Processing And Presentation (GO:0002606)	*CCL21;NOD1*
Positive Regulation Of Endothelial Cell Development (GO:1901552)	*CDH5;CLDN5*
Positive Regulation Of Establishment Of Endothelial Barrier (GO:1903142)	*CDH5;CLDN5*
Regulation Of Basement Membrane Organization (GO:0110011)	*LAMC1;NID1*
Regulation Of Substrate Adhesion-Dependent Cell Spreading (GO:1900024)	*DOCK5;PDPN;EFNA5;TRIOBP*

Rho Protein Signal Transduction (GO:0007266)	*RHOJ;PDPN;ARHGAP29;CELSR1*
Regulation Of Extracellular Matrix Organization (GO:1903053)	*TIE1;LAMC1;NID1*
Regulation Of T Cell Migration (GO:2000404)	*APP;CCL21;CD200*
Negative Regulation Of Programmed Cell Death (GO:0043069)	*KANK2;TJP1;SHC1;FLT4;PDPN;MYO18A;HSPB1;IGF1;HSP90B1;TXNDC5*
Blood Vessel Morphogenesis (GO:0048514)	*TBX1;EGFL7;FLT4;PROX1*
Regulation Of Endothelial Cell Proliferation (GO:0001936)	*EGFL7;FLT4;TIE1;PTPRM;PROX1*
Regulation Of Peptidyl-Serine Phosphorylation (GO:0033135)	*APP;PDE4D;SH2D3C;CNKSR3;PPM1F*
Regulation Of Blood Vessel Endothelial Cell Migration (GO:0043535)	*JUP;HSPB1;PLCG1;EPHA2*
Negative Regulation Of Cellular Process (GO:0048523)	*KANK2;TBX1;APP;PPP1R13B;HYAL2;PDPN;ADAM10;IGFBP7;EMILIN1;PROX1;CLU;CD200*
Regulation Of Extracellular Matrix Assembly (GO:1901201)	*TIE1;EMILIN1*
Positive Regulation Of T Cell Migration (GO:2000406)	*APP;CCL21;ADAM10*
Axon Guidance (GO:0007411)	*ROBO4;UNC5B;NFIB;PTPRM;EFNA5;KALRN*
Regulation Of Apoptotic Process (GO:0042981)	*SHC1;FLT4;HSPB1;IGF1;CLU;DUSP6;HSP90B1;TJP1;PDPN;MYO18A;EMILIN1;CCNL2;BCAR1;TXNDC5*

Negative Regulation Of Macrophage Migration (GO:1905522)	*EMILIN1;CD200*
Lens Fiber Cell Development (GO:0070307)	*PROX1;EPHA2*
Positive Regulation Of Vasculature Development (GO:1904018)	*CDH5;JUP;HSPB1;EMILIN1;PLCG1*
Regulation Of Chemotaxis (GO:0050920)	*CCL21;NOVA2;PPM1F*
Negative Regulation Of Wound Healing (GO:0061045)	*MMRN1;TFPI;PHLDB2*
Establishment Of Endothelial Barrier (GO:0061028)	*TJP1;ROBO4;PDE4D*
Vascular Endothelial Growth Factor Receptor Signaling Pathway (GO:0048010)	*FLT4;HSPB1;BCAR1*
Heterotypic Cell-Cell Adhesion (GO:0034113)	*JUP;ITGA5;CD200*
Positive Regulation Of Locomotion (GO:0040017)	*CAVIN1;CCL21;PPM1F*
Regulation Of Dendritic Cell Antigen Processing And Presentation (GO:0002604)	*CCL21;NOD1*
Regulation Of Macrophage Migration (GO:1905521)	*MTUS1;EMILIN1*
Positive Regulation Of Extracellular Matrix Assembly (GO:1901203)	*EMILIN1;PHLDB2*
Glutamine Transport (GO:0006868)	*SLC38A1;SLC38A2*
Monocyte Activation (GO:0042117)	*HYAL2;DYSF*

Nervous System Development (GO:0007399)	*APP;NCKAP1;DCTN1;TMOD2;PROX1;CELSR1;EFNA5;RAPGEF5;KALRN;SPTBN1*
Positive Regulation Of Cellular Process (GO:0048522)	*TBX1;TJP1;CLDN5;CCL21;CAVIN1;SHC1;FLT4;ADAM10;IGF1;LAMC1;PROX1;NID1*
Regulation Of Sprouting Angiogenesis (GO:1903670)	*TJP1;RHOJ;ITGA5*
Negative Regulation Of Leukocyte Migration (GO:0002686)	*EMILIN1;CD200*
Regulation Of Actin Filament-Based Movement (GO:1903115)	*PDE4D;PDPN*
Negative Regulation Of Supramolecular Fiber Organization (GO:1902904)	*CDH5;EMILIN1;CLU;KANK3*
Cell-Cell Junction Assembly (GO:0007043)	*TJP1;CDH5;CLDN5;JUP*
Positive Regulation Of GTPase Activity (GO:0043547)	*DOCK6;CCL21;DOCK9;ARHGAP29;RAPGEF5;EPHA2;RASGRP3*
Positive Regulation Of Intracellular Signal Transduction (GO:1902533)	*TBX1;APP;NFAT5;RBPMS;CCL21;UNC5B;SHC1;FLT4;DHX15;NOD1;IGF1*
Positive Regulation Of Hydrolase Activity (GO:0051345)	*DOCK6;CCL21;DOCK9;RAPGEF5;CD200;RASGRP3*
Extracellular Matrix Organization (GO:0030198)	*APP;FLRT2;PXDN;ADAM10;LAMC1;NID1*
Macrophage Activation (GO:0042116)	*APP;DYSF;CLU*
Positive Regulation Of Protein Phosphorylation (GO:0001934)	*TBX1;APP;RBPMS;CCL21;FLT4;SH2D3C;IGF1;ITGA5;EFNA5*

		Regulation Of Glycoprotein Biosynthetic Process (GO:0010559)	*CCL21;IGF1*
		Neuron Projection Maintenance (GO:1990535)	*APP;DCTN1*
		Microtubule Anchoring At Centrosome (GO:0034454)	*DCTN1;NINL*
		Negative Regulation Of Sequestering Of Calcium Ion (GO:0051283)	*CCL21;PLCG1;CLU*
		Enzyme-Linked Receptor Protein Signaling Pathway (GO:0007167)	*FLT4;TIE1;PILRB;BCAR1;EPHA2*
		Protein Dephosphorylation (GO:0006470)	*PTPRE;PTPRM;PPP2R5A;DUSP6;PPM1F*
		Positive Regulation Of Endothelial Cell Proliferation (GO:0001938)	*EGFL7;FLT4;PROX1;PLCG1*
		Positive Regulation Of Endocytosis (GO:0045807)	*CCL21;SGIP1;BICD1;CLU*
		Regulation Of Protein Tyrosine Kinase Activity (GO:0061097)	*APP;SHC1;HYAL2*
		Release Of Sequestered Calcium Ion Into Cytosol (GO:0051209)	*CCL21;PLCG1;CLU*
		Circulatory System Development (GO:0072359)	*TBX1;CDH5;FLT4;ALPK3;HSPG2*
		Regulation Of Protein Dephosphorylation (GO:0035304)	*CDH5;PPP2R5A;HSP90B1*
Mast cells			
Fc epsilon RI signaling pathway	*ALOX5;RAC2;FCER1A;MS4A2*	Cytokine-Mediated Signaling Pathway (GO:0019221)	*IL1RL1;FER;KIT;CTSG;IL18R1;RHEX*

Arachidonic acid metabolism	*HPGDS;ALOX5;PTGS1*	Prostaglandin Metabolic Process (GO:0006693)	*HPGDS;HPGD;PTGS1*
Renin-angiotensin system	*CPA3;CTSG*	Leukocyte Aggregation (GO:0070486)	*RAC2;CD44*
Serotonergic synapse	*ALOX5;SLC18A2;PTGS1*	Lipoxygenase Pathway (GO:0019372)	*HPGD;ALOX5*
Sphingolipid signaling pathway	*RAC2;FCER1A;MS4A2*	Angiotensin Maturation (GO:0002003)	*CPA3;CTSG*
Asthma	*FCER1A;MS4A2*	Regulation Of Angiotensin Levels In Blood (GO:0002002)	*CPA3;CTSG*
Phospholipase D signaling pathway	*KIT;FCER1A;MS4A2*	Actin Cytoskeleton Reorganization (GO:0031532)	*FER;KIT;TNIK*
		Mast Cell Activation Involved In Immune Response (GO:0002279)	*KIT;FCER1A*
		Mast Cell Degranulation (GO:0043303)	*KIT;FCER1A*
		Mast Cell Mediated Immunity (GO:0002448)	*KIT;FCER1A*
		Regulation Of Lamellipodium Organization (GO:1902743)	*FER;CD44*
		Myeloid Leukocyte Differentiation (GO:0002573)	*IL1RL1;KIT;LIF*
		Prostanoid Metabolic Process (GO:0006692)	*HPGDS;HPGD*
		Regulation Of MAPK Cascade (GO:0043408)	*GRAP2;KIT;LIF;TNIK*
		Macrophage Differentiation (GO:0030225)	*IL1RL1;LIF*
		Negative Regulation Of Intrinsic Apoptotic Signaling Pathway In Response To DNA Damage (GO:1902230)	*CLU;CD44*

Fc Receptor Signaling Pathway (GO:0038093)	*FER;KIT*
Regulation Of Reactive Oxygen Species Biosynthetic Process (GO:1903426)	*ALOX5;SLC18A2*
Positive Regulation Of DNA-binding Transcription Factor Activity (GO:0051091)	*FER;KIT;CLU;IL18R1*
